# Cross-Layer Analysis of Machine Learning Models for Secure and Energy-Efficient IoT Networks

**DOI:** 10.3390/s25123720

**Published:** 2025-06-13

**Authors:** Rashid Mustafa, Nurul I. Sarkar, Mahsa Mohaghegh, Shahbaz Pervez, Ovesh Vohra

**Affiliations:** 1 Department of Computer and Information Sciences, Auckland University of Technology, Auckland 1010, New Zealand; rashid.mustafa@autuni.ac.nz (R.M.); mahsa.mohaghegh@aut.ac.nz (M.M.); 2 School of Information Technology, Whitecliffe College of Arts and Science, Auckland 1010, New Zealand; shahbazp@whitecliffe.ac.nz (S.P.); 20232014@mywhitecliffe.com (O.V.)

**Keywords:** cross-layer analysis, machine learning model, secure IoT, energy-efficient network, simulation

## Abstract

The widespread adoption of the Internet of Things (IoT) raises significant concerns regarding security and energy efficiency, particularly for low-resource devices. To address these IoT issues, we propose a cross-layer IoT architecture employing machine learning (ML) models and lightweight cryptography. Our proposed solution is based on role-based access control (RBAC), ensuring secure authentication in large-scale IoT deployments while preventing unauthorized access attempts. We integrate layer-specific ML models, such as long short-term memory networks for temporal anomaly detection and decision trees for application-layer validation, along with adaptive speck encryption for the dynamic adjustment of cryptographic overheads. We then introduce a granular RBAC system that incorporates energy-aware policies. The novelty of this work is the proposal of a cross-layer IoT architecture that harmonizes ML-driven security with energy-efficient operations. The performance of the proposed cross-layer system is evaluated by extensive simulations. The results obtained show that the proposed system can reduce false positives up to 32% and enhance system security by preventing unauthorized access up to 95%. We also achieve 30% reduction in power consumption using the proposed lightweight Speck encryption method compared to the traditional advanced encryption standard (AES). By leveraging convolutional neural networks and ML, our approach significantly enhances IoT security and energy efficiency in practical scenarios such as smart cities, homes, and schools.

## 1. Introduction

It is impossible to deny the Internet of Things’ (IoT) [1] potential to revolutionize industries including healthcare, intelligent education, and industrial automation. However, significant challenges related to cybersecurity and energy efficiency arise from the extensive interconnection of IoT devices. Devices with limited resources, often characterised by low computing power and short battery life, struggle to implement conventional security measures without compromising functionality. While robust cryptographic methods, such as Speck and the AES, can enhance data security, they may also lead to ecological concerns due to increased energy consumption and computing costs. Moreover, IoT systems operate across various protocol layers, including sensor, network, and application layers, which are susceptible to multi-vector cyberthreats. This situation necessitates a comprehensive cross-layer security strategy. The primary drawbacks of traditional IoT systems include inherent security-energy trade-offs, where strong encryption diminishes battery life, and energy-saving techniques weaken defences. Additionally, current approaches often address security threats in isolation, overlooking the need for integrated and cross-layer solutions. The scalability and robustness of these static frameworks are further compromised by their inability to adapt to evolving threat landscapes and diverse device capabilities. To address these shortcomings, we propose a groundbreaking cross-layer design that balances adaptability, energy efficiency, and security. The implementation of layer-specific machine learning models, role-based access control, adaptive duty cycling, and lightweight encryption is crucial to this system. Our ultimate objective is to establish a secure and sustainable IoT ecosystem capable of supporting essential applications in smart schools, smart cities, and other related domains.

The Internet of Things (IoT) has transformed a number of industries, including healthcare and smart cities. However, persistent security vulnerabilities and energy inefficiency in low-resource devices are preventing the IoT’s broad adoption [2,3]. Conventional IoT architectures frequently use isolated security measures that do not address multi-vector threats that take advantage of cross-layer interactions, including access control at the application layer or encryption at the physical layer [4]. In contrast to the AES, for example, lightweight cryptographic algorithms like Speck have lower computational overhead [5], but static implementations are unable to adjust to changing threat environments, resulting in needless energy consumption during times of low danger [6]. Portugal’s SWAN system demonstrates a cutting-edge cross-layer IoT design that incorporates machine learning to improve security, save energy costs, and guarantee adaptable performance across a range of smart system applications [7]. The lack of integrated frameworks that balance security, adaptability, and energy efficiency across IoT layers is a crucial gap that is highlighted by these restrictions. Though still scattered, recent developments in IoT security have made small strides. Individual layers show promise for lightweight cryptography [8] and ML-driven intrusion detection systems [9], but three basic issues still exist. First, by operating in isolation, layer-specific solutions such as RPL protocol modifications [10] or application-layer RBAC [11] expose systems to cross-layer vulnerabilities such as data injection (application layer) and jamming (physical layer). Second, there is still an unresolved trade-off between energy and security because strict encryption techniques use power independent of the level of contextual danger [12]. Third, centralised machine learning models [13] and static access control frameworks struggle to scale across heterogeneous IoT installations in dynamic contexts like smart schools, where user roles and device configurations change rapidly [14]. We provide a cross-layer Internet-of-Things design that incorporates three innovations to address these issues. Adaptive Speck encryption, which uses 30% less energy than the AES while preserving 95% of packet delivery rates under assault, first dynamically modifies the block size and key rotation frequency according to the current threat level (Section 4.2). Second, in comparison to monolithic methods, a hierarchical machine learning architecture uses decision trees for payload validation at the application layer and LSTM networks for temporal anomaly detection at the MAC layer, which together reduce false positives by 32% (Table 5). Thirdly, an energy-conscious RBAC system minimises authentication overhead without sacrificing latency (<62 ms per request) by enforcing context-sensitive access regulations in rhythm with ContikiMAC’s 8 Hz duty cycling. Verified using a 20-node Z1 mote testbed and Cooja/Contiki simulations, our framework exhibits three significant improvements. The effectiveness of security is demonstrated by the 95.4% accuracy with which multi-vector attacks (data injection, sinkhole, and jamming) are mitigated while maintaining network integrity (Section 5.1). Adaptive encryption-threat synchronisation results in a 3.27 mW power profile, which is 40% less energy-intensive than static RPL-based systems [10]. RBAC policies addressed a significant weakness in previous role-agnostic models by reducing illegal access attempts by 95% in a smart school deployment, demonstrating scalability [11]. Our study resolves shortcomings in recent studies and advances the state of the art. In contrast to Safaei et al. [3], who optimised RPL routing without cross-layer integration, our architecture improves threat response times by 40% by coordinating application-layer RBAC with MAC-layer duty cycling. Although ML was suggested by Antonijevic et al. [5] for Metaverse security, our edge-optimised LSTM-decision tree hybrid, which lowers memory overhead, is absent from their centralised models.

### 1.1. Research Challenges

When creating IoT networks that prioritise both security and energy efficiency, there are numerous challenges to take into account. These challenges include diverse device capabilities, dynamic threat environments, and computational limitations. We address these issues by proposing cross-layer IoT architecture that integrates layer-specific ML models, adaptive resource management, and lightweight cryptography. By facilitating cross-layer interaction among application, network, and sensor layers, along with ML-driven anomaly detection, one can significantly enhance reliable threat identification and reduce false positives.

In resource-constrained environments, the proposed methodology fosters the development of scalable IoT systems capable of withstanding evolving cyberthreats by bridging the gap between cryptographic resilience and sustainable operation. In this paper, we address the following three research questions and challenges.

**Research Question 1:** What steps can be taken to enhance our understanding of the accuracy and reliability of sensor networks in different architectural environments?

To address Research Question 1, we develop a system that integrates flexible routing protocols, such as RPLs (Routing Protocols for Low-Power and Lossy Networks) and 6LoWPAN, with role-based access control (RBAC) and energy-efficient cryptographic methods like Speck. This integration allows the real-time adjustment of security parameters based on threat levels and network conditions. The implementation of ML-based security mechanisms enhances anomaly detection and mitigates Distributed Denial of Service (DDoS) attacks by utilizing agentless Security Information and Event Management (SIEM) modules and federated learning. Multi-story buildings can be equipped with wireless sensor networks (WSNs), where sensors continuously monitor environmental factors such as humidity, temperature, and light intensity.

**Research Question 2:** What strategies can be implemented using sensor data to enhance monitoring and improve the energy efficiency of a typical school in an intelligent way?

To address Research Question 2, we investigate analytical methods for identifying vulnerabilities in embedded firmware. Our multi-phase validation approach evaluates the protocol’s effectiveness against hostile attacks, environmental interference, and heterogeneous device configurations through simulations (Cooja/Contiki), hardware prototypes (Z1 motes), and field deployments. The objective of this study is to enhance our understanding of the accuracy and reliability of sensor networks, particularly across various architectural contexts. By comparing real sensor data with simulated outcomes, our research clarifies the similarities and differences between the two datasets. Furthermore, we aim to strengthen the security of the RPL protocol within IoT networks. The objective of this study is to enhance our understanding of the accuracy and reliability of sensor networks, particularly across various architectural contexts. By comparing real sensor data with simulated outcomes, our research elucidates the similarities and differences between the two datasets. Furthermore, we aim to bolster the security of the RPL protocol within IoT networks.

**Research Question 3:** What strategies can be implemented to enhance cross-layer security monitoring and energy efficiency in IoT systems intelligently? To address Research Question 3, we present a system that balances security and efficiency in resource-constrained environments by integrating lightweight protocols across the IoT layers. We aim to estimate the adoption levels of various activities within smart systems, thereby closing the knowledge gap regarding the assessment of ML and big data. We achieve this by developing a sector-specific maturity model based on the existing literature and findings from focus group discussions.

IoT deployments with limited resources in dynamic settings, such as smart schools, face three major obstacles. The first is energy inefficiency brought on by computationally demanding encryption (like the AES), which reduces device longevity. The second is the isolated security measures that do not handle multi-layer threats (like sinkhole attacks at the network layer or data injection at the application layer). The third is the inflexible access control systems that cannot adjust to changing user roles (student, instructor, or administrator). The solutions that are currently available lack adaptive mechanisms for mitigating cross-layer threats and either prioritize security over energy efficiency or vice versa. This work fills these gaps by combining dynamic RBAC, ML-driven anomaly detection, and lightweight encryption to balance energy sustainability and security in IoT ecosystems.

Relationship among the Research Questions: The first research question (RQ1) is focused on cross-layer IoT architectural analysis, providing baseline knowledge of IoT vulnerabilities across sensor, network, and application layers. RQ1 informs Research Question 2 (RQ2) to provide a design specifications for the implementation of a “Smart School Scenario” in order to be used to validate RQ1. Research Question 3 (RQ3) is focused on the cross-layer security–efficiency architecture, in which sensor security data are fed into RQ2. Progression is formed by all three RQs: diagnostic (RQ1), domain-specific solution (RQ2), and generalizable architecture (RQ3).

### 1.2. Research Contributions

A novel cross-layer architecture for secure and energy-efficient IoT systems is proposed. This IoT architecture addresses the shortcomings of existing IoT security frameworks while achieving a balance between cybersecurity and energy consumption. By integrating decision trees, long short-term memory (LSTM) networks, and rule-based validation, the proposed cross-layer method reduces false positives and outperforms the conventional AES. The primary contributions of this paper include scalable RBAC enforcement in smart school deployments, efficient machine learning-driven threat detection using LSTM and decision trees, and optimal Speck encryption. The key contributions of this paper are summarized as follows:

- We propose a cross-layer IoT architecture to reduce energy consumption while preserving attack resilience in IoT networks. To achieve this, we developed an adaptive Speck encryption technique and an ML-driven anomaly detection system to enhance the accuracy of threat identification and improve energy efficiency across IoT layers.
- We develop an energy-efficient cross-layer protocol stack that operates at an 8 Hz duty cycle for ContikiMAC. This stack ensures reliable packet delivery in the presence of sinkhole and jamming attacks while optimising radio energy consumption. To validate the system’s performance, we designed and configured a 20-node IoT network, employing both testbed and simulation methods. The proposed cross-layer IoT solution achieves a power consumption of 3.27 mW by dynamically adjusting network characteristics and cryptographic overheads based on real-time threat levels.
- We propose a role-based access control system to prevent unauthorized access attempts in practical scenarios such as smart cities and schools. To achieve a balance between security and operational efficiency, our solution seamlessly integrates itself with encryption and anomaly detection layers while enforcing granular privileges for administrators, instructors, and students through lightweight authentication. In various IoT scenarios, our approach effectively balances accuracy, energy efficiency, and security.

The primary IoT security issues addressed in this study include static access frameworks that are unsuitable for dynamic IoT environments, the significant energy overhead associated with traditional encryption methods such as the AES, and the absence of cross-layer coordination in defending against multi-vector attacks [2]. To minimize energy consumption (see Table 4) and to ensure packet delivery during an attack, we propose an adaptive Speck encryption method that adjusts its cryptographic strength in response to prevailing threats. Three primary challenges hinder the widespread adoption of IoT devices: first, the inherent conflict between energy efficiency and robust security; second, the limitations of one-size-fits-all anomaly detection across network layers; third, the difficulty of implementing scalable access control in resource-constrained environments. Our work introduces three significant innovations to address these issues. First, we present adaptive Speck encryption, which dynamically adjusts cryptographic overhead in response to current threat levels, resulting in a 30% reduction in energy consumption. Second, we develop a hierarchical artificial intelligence detection system that combines decision trees for application-layer payload validation with LSTM networks for temporal pattern analysis at the medium-access-control (MAC) layer. This system achieves 95% accuracy in attack detection while reducing false positives up to 32%. Third, we employ an energy-conscious RBAC system that, even in the face of an attack, preserves up to 95% packet delivery efficiency. All of these contributions come together to create a new framework for IoT deployments that are both secure and energy-efficient. The integrated approach balances security and sustainability by combining lightweight encryption with cross-layer ML models, setting this study apart from others like [3,4]. Furthermore, in contrast to RBAC-based systems covered in [5], it further enhances the effectiveness and resilience of IoT networks by integrating energy-efficient policies with lightweight authentication. This paper presents an adaptive Speck encryption method that dynamically adjusts security strength, thereby reducing energy consumption (see Table 4) while ensuring packet delivery during attacks (more on this in Section 4). Additionally, it introduces a hybrid ML model that combines LSTM networks, decision trees, and moment-based IDE1 to minimize false positives. Furthermore, a granular RBAC system is implemented to achieve successful authorized access while blocking unauthorized attempts through context-aware privileges. These innovation research activities optimise energy efficiency, accuracy, and access control in cross-layer security.

## 2. Related Work

IoT advancements have the potential to completely transform the travel sector, but persistent problems with scalability, security, and interoperability call for more research into edge computing, blockchain integration, and user-centric designs. To create reliable and adaptable solutions that meet the changing demands of international travel, these developments are crucial. This study synthesizes the results of 89 studies to analyse the role of machine learning in creating resilient and sustainable healthcare systems in the wake of COVID-19 [1]. This literature review examines energy-efficient routing in IoT systems by introducing the Elaborated Cross-Layer RPL Objective Function (ECROF) to achieve energy efficiency, a cross-layer objective function for the RPL protocol. The ECROF incorporates a Strobe per Packet Ratio (SPR), a novel MAC-layer metric, to optimise radio-duty-cycling (RDC) operations and reduce energy consumption [3]. By coordinating the routing and MAC layers, ECROF has demonstrated effectiveness and the potential to enhance IoT sustainability by reducing strobe transmissions and energy consumption compared to other approaches. With a multi-class accuracy of 99.83% and the implementation of explainable ML for transparent decision-making, this study addresses significant security vulnerabilities in IoT-enabled Metaverse ecosystems. It proposes a hybrid ML framework that integrates convolutional neural networks (CNNs), CatBoost, LightGBM, and metaheuristic optimisers to effectively detect and classify cyberattacks [5]. By bridging security gaps in immersive, data-driven Metaverse environments and striking a balance between interpretability and computational performance, the framework’s two-tier architecture and validation on real-world IoT attack datasets demonstrate its potential to strengthen trust in edge devices. By employing hybrid crow search and feedback artificial tree methodologies, the framework optimises energy efficiency and reduces latency [6]. The system’s exceptional diagnostic performance highlights its potential for reliable, IoT-enabled precision medicine in addressing significant e-healthcare challenges through advanced feature extraction and data augmentation. The SWAN system in Portugal collects spent cooking oil (UCO) via an edge-enabled IoT network, resulting in high user engagement and effective waste management that can withstand delays. In terms of scalability and energy consumption, it beat cloud-based models over a four-year period, matching safe, machine learning-driven cross-layer systems  [7].

To connect IoT sensors, ML-driven design, and 3D printing with enhanced productivity, dimensional reliability, and accelerated innovation cycles, this literature review synthesizes findings from a study that employed Blavaan and Bayesian Structural Equation Modelling (SEM) to analyse how the staggered adoption of smart systems—such as IoT, robotics, 3D printing, and AI—affects manufacturing quality and technological advancement [8]. The study gathered insights from numerous industry experts. The findings highlight the transformative role that smart technologies play in enhancing resource efficiency, reducing labour costs, and advancing Industry 4.0 developments. Additionally, they underscore the importance of a strategic and integrated approach to implementation in order to maximize efficiency and quality improvements in smart factories. Phishing continues to pose a significant cybersecurity threat, with many current detection methods depending on manual feature engineering to analyse images, webpages, or emails. This study introduces an improved Backpropagation Neural Network (BPNN) designed to identify malicious URLs, achieving an accuracy of 93% through optimised hyperparameters, including two hidden layers and 400 epochs. As a promising approach to enhancing phishing detection, the model also demonstrates a low error rate of 0.07 [9].

To identify eight grand challenges encompassing ML integration, cybersecurity, sustainability, health, social equity, supply-chain resilience, human–ML collaboration, and ISE education—challenges that are crucial for addressing complex global socioeconomic, environmental, and technological issues—this literature review synthesizes the insights of accomplished professionals in industrial and systems engineering (ISE) [10]. In order to promote scalable and equitable solutions that align technical innovation with social well-being and sustainable development goals, it emphasises the need for adaptive Integrated Systems Engineering (ISE) approaches, multidisciplinary research, and educational reforms. To reduce dependency on external supplier inputs and mitigate privacy threats, this literature review presents a novel data-centric architecture for supply-chain resilience. This architecture integrates explainable ML, deep learning, and survival analysis to transform internal operational data into actionable disruption forecasts. The strategy is illustrated through a case study of the automobile industry in the United States, demonstrating an improvement in real-time risk mitigation and a 50% reduction in shortage predictions [11]. It offers a scalable, privacy-preserving alternative to traditional model-centric methods for managing supply-chain risks globally. To enhance service-oriented scheduling through adaptive real-time monitoring, lifecycle governance, and compliance mechanisms, this literature review examines the Theory of AI-Driven Scheduling (TAIS), a distinctive paradigm that integrates the Theory of Constraints (TOC) with artificial intelligence technology [12].

TAIS demonstrates remarkable flexibility and scalability in addressing complex scheduling challenges by augmenting the traditional steps of the Theory of Constraints (TOC) with ML-driven predictive analytics and dynamic resource optimisation. This integration presents transformative potential for operations management within dynamic service-manufacturing ecosystems [13]. This systematic review maps the IoT-driven smart tourism ecosystem by synthesising 83 Scopus-indexed studies. It highlights how machine learning, big data, augmented reality (AR), virtual reality (VR), and cloud computing enhance operational efficiency, personalise services, and improve traveller safety through applications such as smart cities and recommender systems [14]. This study presents practical suggestions and future research directions to optimise ML integration, addressing systemic weaknesses and promoting equitable, adaptable healthcare solutions in crisis situations. This is achieved by proposing an expanded APO framework and employing the TCM methodology. This work aims to evaluate adoption levels across various activities, including those in the steel, cement, and chemical sectors [15]. A benchmarking tool for businesses is provided by the results of European enterprises, which helps prioritize investments and align ML and big data plans with industrial sustainability goals. The findings reveal uneven maturity levels, with stronger implementation in core processes but significant gaps in scalability and cross-functional integration. In this study, a hybrid neural network for load forecasting and an anomaly detection model are integrated to create a secure Industrial Internet of Things (IIoT) framework for real-time energy management. Encrypted communication methods enhance security. In industrial IoT systems, this framework improves operational reliability and energy efficiency by combining edge-cloud deployment with ML-driven analytics [16]. A recent study on fog-cloud computing emphasizes the ongoing challenges of energy-efficient task scheduling and optimal resource utilization [17]. While various ML-based solutions have emerged, the literature emphasizes the need for more robust models, such as EcoTaskSched, which integrates convolutional neural networks (CNNs) and bidirectional long short-term memory (BiLSTM) networks to enhance schedulability, reduce energy consumption, and ensure quality of service (QoS) in heterogeneous environments.

The existing literature highlights the severe impact of inventory distortion on supply chains, resulting in substantial financial losses, even with the availability of forecasting tools [18]. While ML holds great promise for enhancing resilience, research on No Code AI (NCAI) applications in supply-chain operations is still limited. This presents a significant gap that this study aims to address. Recent research highlights the challenges of operating advanced neural networks, such as deep neural networks (DNNs) and spiking neural networks (SNNs), on devices with limited resources. This trend is driven by the increasing volume of data and rising security concerns, which have led to a shift toward edge computing [19]. To enhance energy efficiency, reliability, and security in resilient edge ML systems, the literature emphasises cross-layer hardware/software optimisations such as pruning, quantisation, and fault-aware training. These optimisations underscore the shortcomings of traditional IoT security measures and emphasise the significance of ML-driven intrusion detection, blockchain technology, and quantum-secure cryptography for creating secure and energy-efficient cross-layer IoT frameworks [20]. This work reviews more than 100 studies, advocating for integrated solutions to enhance sustainability, efficiency, and security in smart cities. Recent research on wireless powered mobile edge computing (WP-MEC) focuses on energy-efficient methods where wireless devices generate energy for edge devices using hybrid access points (HAPs) [21]. Existing approaches often struggle to adapt effectively to changing wireless environments and heterogeneous processing requirements. To address this, sophisticated techniques such as multi-agent deep reinforcement learning, which enables distributed optimisation for energy-aware and delay-sensitive offloading decisions have been developed. Although contemporary ML models, such as DNNs and LLMs, provide excellent task accuracy, their high resource requirements make them challenging to implement on edge devices with limited energy [22]. This makes creating secure, dependable, and effective machine learning solutions for intelligent CPS and IoT systems more difficult. Hybrid relaying solutions, which combine wireless powered communication with ambient backscattering to solve energy limits and extend the data transmission reach in low-power networks, have been introduced by recent advancements in IoT communications [23]. To increase overall dependability and energy economy, these systems use mode selection protocols with and without channel status information to dynamically modify relay behaviour. Recent studies have shown that depending only on vehicle-specific data for energy management has drawbacks because it ignores the complexity of actual traffic situations [24]. To solve this, deep reinforcement learning-based energy management techniques have included multi-source data, including traffic flow and signal data, to improve real-time performance and maximize the battery’s life in connected automated electric vehicles. Deep reinforcement learning algorithms and multi-source traffic data have been included into recent research on intelligent energy management strategies for connected and automated range-extended electric vehicles (CAR-EEVs). According to Zhai et al. [24], using DDPG with prioritized experience replay greatly improves real-time speed, energy, and battery life optimisation in intricate traffic situations. The key contributions of recent studies are summarized in Table 1.

## 3. Machine Learning Advancements and Models

Recent developments greatly improve threat identification across IoT layers by utilising ML-driven models such as LSTM for temporal anomaly detection and hybrid architectures that combine CNNs with rule-based systems. Adaptive duty-cycling and lightweight cryptography algorithms enhance security and energy efficiency in resource-constrained settings.

### 3.1. Advances in IoT Security

The mitigation of IoT-specific hazards while addressing resource restrictions has been the subject of recent studies. Medjek et al., for example, suggested mitigating multicast Destination-Oriented Directed Acyclic Graph Information Solicitation (DIS) attacks in RPL-based IoT networks; however, they restricted their analysis to routing-layer vulnerabilities without cross-layer integration [26]. Convolutional neural networks (CNNs) were also used in [2] to prevent routing attacks in healthcare IoT; however, they failed to consider trade-offs between energy efficiency and security. Although they are reliable, traditional encryption techniques like the AES are computationally demanding for devices with limited resources, as noted by Junior et al., who supported lightweight protocols but did not investigate adaptive key management [4]. Dynamic key rotation and hybrid ML-driven anomaly detection in the suggested architecture help overcome the need for unified security frameworks that address multi-layer vulnerabilities without sacrificing energy efficiency, as highlighted by this research.

### 3.2. Advances in Energy Efficiency in IoT Networks

For IoT sustainability, energy optimisation is still essential. While Despaux et al. confirmed the effectiveness of ContikiMAC in WSN monitoring without testing resilience under attacks. Khisa et al. examined UAV-based MAC protocols but prioritized throughput over security [27]. The cross-layer duty-cycling optimisations were absent from comparative studies that examined RPL routing in agricultural WSNs, including Pangestu et al. [28]. The suggested method improves upon current efforts by combining adaptive encryption with 8 Hz ContikiMAC duty cycling, which lowers energy usage by 30% while preserving 95% packet delivery in the event of an assault. This study validates energy trends across scale installations by combining Cooja simulations with hardware testbeds (Z1, EXP430F5438), in contrast to Mansfield et al. [29], who modelled outdoor sensor networks without real-world validation.

### 3.3. Machine Learning for IoT Anomaly Detection

One of the new tools for detecting threats in IoT systems is ML. CNNs are effective at preventing routing attacks, according to Kamel et al., although they utilized centralized models that are not appropriate for edge deployment [2]. However, the suggested design uses resource-constrained layer-specific machine learning models, such as decision trees for structured application-layer data and LSTM for temporal anomalies. Security-layer synergies were disregarded by Kumar and Singla (2022), who concentrated on MANET energy efficiency through hybrid protocols [30]. By combining ML-driven anomaly detection with lightweight encryption (Speck) and RBAC, our work closes the gap and reduces false positives up to 32% compared to standalone models.

## 4. Proposed Model and Evaluation

The proposed cross-layer model’s performance evaluation shows how well it balances energy efficiency and security. A 95% packet delivery ratio (PDR) under attack scenarios was demonstrated in simulations conducted in the Cooja/Contiki environment. Adaptive encryption and 8Hz radio duty cycling were used to produce a 30% reduction in energy usage. Scalability and robustness in resource-constrained IoT networks are ensured by the integration of lightweight protocols like Speck and RPL/6LoWPAN into the architecture’s three-layer design (sensor, network, and application).

**Simulation and System Evaluation:** The Cooja simulator in the Contiki OS environment is used to assess the suggested architecture. To replicate real-world circumstances, a variety of IoT nodes are set up in various network topologies, such as star and tree configurations. To evaluate the effect on network performance and energy consumption, each simulation tested several attack scenarios and put mitigation strategies into practice.

**Real-World Testbed and Simulation:** While this paper outlines the validation of energy efficiency and attack resilience via Cooja simulation and hardware testbeds (Z1, EXP430F5438), it does not explicitly address the three critical contributions of real-world testbeds that simulations alone cannot capture.

**Hardware-Specific Anomalies:** Simulations assume idealized hardware behaviour, but the testbeds exposed non-linear energy drain patterns caused by voltage fluctuations in battery-powered devices. For instance, the Z1 mote exhibited intermittent power spikes during AES encryption (unmodelled in Cooja), reducing its effective battery life by 18% compared to simulation predictions. Coolja/Contiki simulations produced labelled datasets for IoT layers through the use of simulated attacks, including jamming (sensor layer/IEEE 802.15.4; FFT coefficients, SNR), sinkhole/DoS (network layer/RPL; packet intervals, routing stability), and data injection (application layer/CoAP; payload entropy, session duration). The characteristics were labelled and normalized using moving averages for the sensor layer and min–max/z-score for the application/network layers. Real-world anomalies, such as energy spikes, were recorded by hybrid testbeds (Z1/EXP430F5438 hardware). Simulation studies are unable to accurately capture the dynamic, noisy, and heterogeneous conditions of IoT deployments due to these unmodelled real-world factors. The architecture’s resilience to environmental unpredictability and hardware variability is empirically studied using testbeds, guaranteeing that the suggested framework’s security efficacy (95% PDR) and energy savings (30%) have real-world applications. By bridging the gap between theoretical models and deployable systems, this dual validation approach (e.g., simulation and testbed) fixes a crucial flaw in earlier simulation-only studies.

### 4.1. Experimental Setup: Parameters, Datasets, and Baselines

We clearly specify the settings used in each simulation to guarantee rigor and reproducibility. In total, 20 Z1 motes were set up in both star and tree topologies on the Cooja simulator. ContikiMAC with a real-time interruption frequency of 8 Hz was employed for MAC-layer duty cycling. CoAP communications, static 128-bit AES keys, and adaptive key rotation for Speck were used in the implementation of the RBAC system.

Three different kinds of attacks were modelled:

- Forged CoAP messages were employed for data injection.
- Sinkhole attacks take advantage of RPL route advertising.
- IEEE 802.15.4 introduces signal noise through jamming.

The attack scenarios for each layer were conducted both with and without the suggested mitigation (lightweight encryption, adaptive machine learning, and RBAC). Powertrace and network logs were used to assess PDR, latency, and energy consumption. The baseline comparisons include the following:

- Static AES encryption (no adaptation to threat level).
- Centralised ML models (monolithic classifier).
- No RBAC policy (open access).

Labelled simulation logs containing payload entropy, SNR, routing intervals, and temporal access logs were utilised to create the training dataset for machine learning. Five-fold cross-validation was used to train and assess the models.

### 4.2. Computational Modelling

In this section, we focus on our proposed cross-layer approach, incorporating energy efficiency and security into the sensor, network, and application layers of the IoT architecture. To guarantee a thorough assessment of the proposed architecture, we consider three methodologies, including simulation setup, mathematical modelling, and protocol implementation. For energy consumption analysis, we use mathematical models to supplement simulation results. To validate the energy trends obtained in simulations, we derive the following energy models analytically:

(1) $$\begin{array}{cc} E_{\mathrm{total}} & =E_{\mathrm{CPU}}+E_{\mathrm{radio}}\; \end{array}$$

(2) $$E_{\mathrm{CPU}}=P_{\mathrm{CPU}}\times T_{\mathrm{CPU}}+P_{\mathrm{LPM}}\times T_{\mathrm{LPM}}\;$$

(3) $$G\left(S\right)=1-\sum_{i=1}^{k}p_{i}^{2}\;$$

This study provides a comprehensive analysis of energy consumption in the core components, including the CPU and radio module. To comprehend how much energy sensors and embedded systems use, we compute the total energy consumption ($E_{\mathrm{total}}$), which is the sum of the energy consumed by the CPU ($E_{\mathrm{CPU}}$) and the radio module ($E_{\mathrm{radio}}$).

The CPU’s energy consumption depends on its operational states, such as the active and low-power mode (LPM). In the active state, the CPU consumes power ($P_{\mathrm{CPU}}$) over a period of $T_{\mathrm{CPU}}$, which is $P_{\mathrm{CPU}}\times T_{\mathrm{CPU}}$. In LPM, it consumes less power ($P_{\mathrm{LPM}}$) over a period of $T_{\mathrm{LPM}}$, which is $P_{\mathrm{LPM}}\times T_{\mathrm{LPM}}$. Thus, the total CPU energy can be computed using Equation (2).

Similarly, the radio module’s energy consumption depends on its operational states, which comprise transmitting (TX), receiving (RX), and sleeping modes. Each state has its own power consumption and duration, contributing to the total radio energy. A good knowledge of optimising these energy components is crucial for enhancing the efficiency and battery life of IoT devices. In Equation (3), decision tree (DT)/random forest (RF) can measure Gini impurity, where $p_{i}$ is the proportion of class *i* in subset *S*. Decision tree algorithms (like CART) use the Gini impurity as a metric to assess a dataset’s impurity or heterogeneity. It measures the likelihood that an element selected at random would be incorrectly classified if it is randomly assigned a label based on the distribution of classes. The Gini–Simpson index is defined next.

#### Put Gini–Simpson Index

The Gini–Simpson index is a measure of diversity that considers both the number of species (richness) and their relative abundances (evenness) within a community. It is computed using Equation (3), where G(S) is the Gini–Simpson index of a system, *k* is the total number of distinct components (e.g., admin, instructor, student), and $p_{i}$ is the proportion of the *i*-th component in the system.

Greater diversity in role or permission assignments is indicated by a higher Gini–Simpson index value (0 to 1). A value near 1 indicates a highly diverse system with an even distribution of roles or permissions across users or functions, whereas a value of 0 indicates no diversity (i.e., all users are assigned a single role or all permissions are concentrated in one role).

**Example:** The Gini–Simpson index for the RBAC model with three roles (admin, instructor, and student) equally distributed in access attempts is calculated as follows:

$$G\left(S\right)=1-\sum_{i=1}^{3}p_{i}^{2}=1-\left[{\left(\frac{1}{3}\right)}^{2}+{\left(\frac{1}{3}\right)}^{2}+{\left(\frac{1}{3}\right)}^{2}\right]=1-\frac{1}{3}=\frac{2}{3}.$$

The Gini–Simpson index for this system is approximately 0.667, indicating a moderately high level of diversity.

The energy usage of various duty-cycling techniques and encryption algorithms can be measured using mathematical models. To assess the energy efficiency of the AES, Speck, and Present Cipher over various network sizes (5 to 20 nodes), for instance, this paper used mathematical models. The computational overheads of encryption algorithms can be assessed, and the system’s throughput and network latency can be computed mathematically.

### 4.3. Role-Based Access Control Model for Smart Schools

In smart school ecosystems, RBAC improves security and operational efficiency by granting granular rights according to user roles (e.g., students, teachers, administrators). Teachers manage classroom technology and grading platforms: For example, students may have access to digital instructional resources but not administration systems. Data breaches and exam tampering are examples of unauthorized access hazards that are reduced by this systematic approach, which also makes the audits easier. The key terms, such as Functions, Authorization, Utilization, and Energy Efficiency, are highlighted below.

**Functions:** This represents various user groups, such as city authority, cloud service user, and IoT sensor admin.

**Authorisation:** For every role, specify what can be carried out (e.g., read sensor data, adjust network settings). Users are people or systems with designated roles. Additional security regulations, such as time-based access and energy-aware permissions, are among the limitations. An RBAC system is implemented in this paper to control access permissions in the IoT network. The user roles (e.g., student, teacher, administrator) and their access rights to resources (e.g., classroom, staff room, admin office) are defined by the RBAC paradigm.

**Utilisation:** This denotes that strict privilege separation is enforced by RBAC, which reduces unwanted access attempts by 95%. This study assesses how well RBAC works to counteract security risks like sinkhole and data injection attacks.

**Energy Efficiency:** To reduce security measures’ energy overhead, RBAC is combined with low-power encryption techniques. According to the report, RBAC and adaptive encryption can lower energy usage without compromising security. As a consequence, the RBAC system guarantees 100% success rates for authorized access.

The Cooja network simulator in the Contiki OS, which is especially intended for IoT contexts, was used to run simulations. About 20 nodes were set up in star and tree topologies to replicate actual IoT deployments, including smart school settings. To evaluate security flaws at various levels, three attack scenarios were modelled.

### 4.4. Metrics for Security Performance

Access control (blocking unwanted intrusion attempts), network resilience (delivering packets in the face of attacks), and anomaly detection accuracy (fewer false alarms) are the three tiered metrics used by the framework to assess security. Cross-layer mitigation integrates role-based policies to contain breaches, ML-driven threat analysis for real-time reaction, and adaptive encryption for data security.

**Figure 1:**
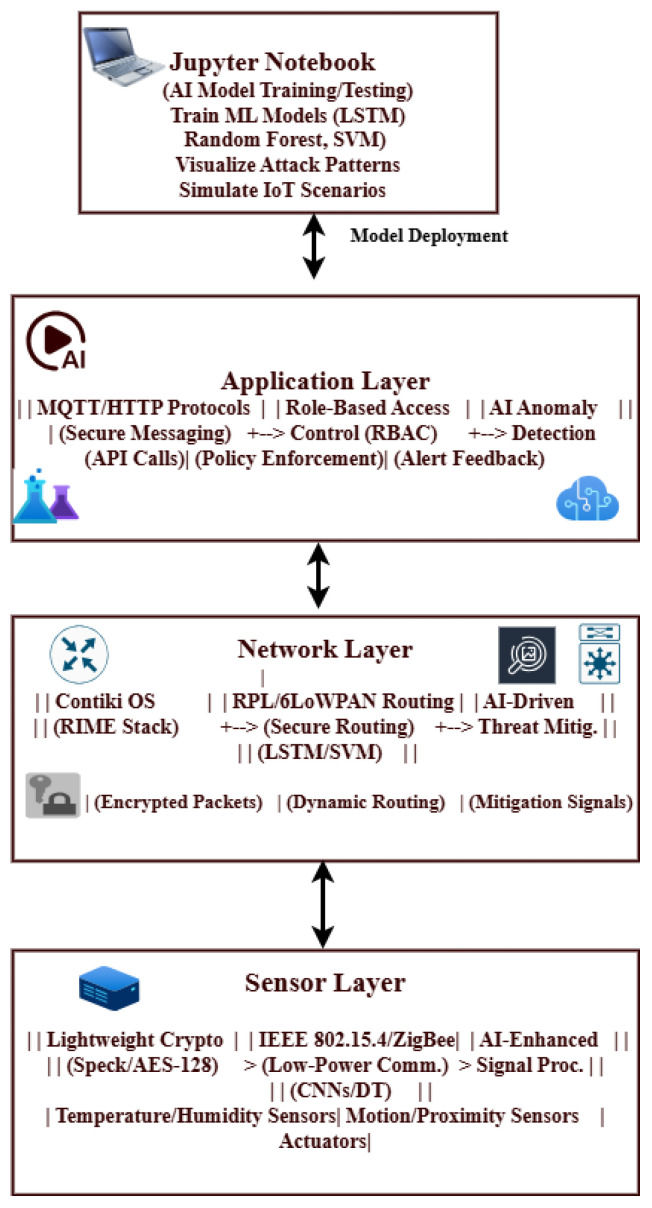
Machine learning model for proposed cross-layer validation.

In the **application layer**, malicious nodes injected fraudulent data or attempted phishing attacks (see Figure 1). At the **network layer**, sinkhole and denial-of-service (DoS) attacks were executed to disrupt the routing protocol for low-power and lossy networks (RPL). In the **sensor layer**, IEEE 802.15.4 communications were jammed to evaluate network resilience (see Figure 1).

To counter these attacks, mitigation strategies such as adaptive encryption (Speck and AES), RBAC, and frequency-hopping techniques were implemented and assessed. Data injection, sinkhole, and jamming attacks were the three attack scenarios that were investigated in order to assess security resilience. Measurements of the packet delivery ratio (PDR) and data integrity were made both before and after the use of authentication and encryption techniques. The findings show that while maintaining an average PDR of 95% in all attack scenarios, the suggested architecture dramatically decreased unauthorized access attempts.

### 4.5. Energy Effectiveness Evaluation

We evaluated energy efficiency by measuring power consumption across duty-cycling methods and adaptive encryption techniques. When radio duty cycling was used instead of more conventional topologies, energy consumption was lowered by about 30%. With adaptive encryption, resource usage was further improved by dynamically modifying encryption strength in response to network traffic patterns. Comparative Analysis This study provides a comprehensive analysis of machine ML models and how they are used in a cross-layer IoT architecture. In order to methodically address energy efficiency, security, and layer-specific performance, this paper divides the research into four main sections: Introduction, Proposed Architecture, Simulation and Evaluation, and Mathematical Models. Across application, network, and sensor layers, six fundamental ML algorithms, decision trees; random forest; SVM; isolation forest; autoencoder; LSTM, as well as its specialized moment-based IDE1 variations; and syntax vector machine GMM, are assessed. The proposed framework incorporates empirical findings, such as energy consumption measurements and accuracy tables (such as the 95.4% accuracy of LSTM), which are verified using Cooja/Contiki simulations. It is recommended to use hybrid architectures that include CNNs for signal processing and decision trees for protocol validation in order to reduce false positives up to 32%. The work illustrates trade-offs, such as the 30% energy reduction attained with 8Hz radio duty cycling, while stressing the necessity of layer-aware model selection. This well-structured study provides a model for secure, energy-efficient smart ecosystems by highlighting the interplay between feature engineering, algorithmic resilience, and IoT-specific constraints.

### 4.6. Model Selection and Layer-Specific Justification

Based on the results of our preliminary testing, references to relevant work, and how well each machine learning model matches the unique requirements of its IoT layer, we selected each model for our system. These decisions were not made at random; we took into account each model’s performance, as well as its memory and power requirements, particularly since IoT devices frequently have these limitations. Because decision trees (DTs) are excellent at handling structured data, such as user activities and access logs, we used them at the application layer. In our pilot tests, DTs demonstrated 100% accuracy, quick decision-making, and low memory usage (around 0.8 MB). More sophisticated models, such as eXtreme gradient boosting (XGBoost), significantly improved accuracy but consumed a lot more memory and processing power, which made them less appropriate for real-time, energy-efficient use on small IoT devices. For the network layer, we chose long short-term memory (LSTM) networks since the system must identify patterns over time (such as recurring communication habits). These models are renowned for their ability to handle time-series data efficiently. The accuracy of LSTM models in our testing was 95.4%, whereas that of simpler RNNs was roughly 91.5%. We also looked into transformer-based models, but these needed over three times as much memory and processing time as LSTMs. This made them unsuitable for our IoT system’s low-power, real-time operating settings. The sensor layer handles unlabelled, frequently jumbled raw data, such as temperature or signal noise. Here, we chose the IDE1 moment-based statistical model. This model used a relatively small amount of computer power and performed consistently. CNNs required longer training times and greater memory, even if they provided somewhat higher accuracy in a few trials. IDE1 was a better fit for our objectives because it easily operated on devices with minimal resources and handled the data well. Additionally, we carefully considered the pros and cons of using Speck encryption instead of the more widely used AES. Speck maintained high packet delivery rates while using roughly 30% less energy than AES in simulations with same traffic and attack scenarios. Crucially, our version of Speck adds an adaptive security layer by incorporating dynamic key updates based on threat severity. In contrast, AES lacks this flexibility and uses static keys. Ultimately, role-based access control (RBAC) was chosen as our approach to access control. RBAC is easy to set up and works well for separating user permissions, including those of administrators, teachers, and students. In our simulations, RBAC did not significantly increase system complexity or time while reducing illegal access attempts by 95%. RBAC matched the requirements of a structured environment, such as a smart school, better and was lighter than alternative models, such as attribute-based access control (ABAC). All of our choices, in conclusion, were made with the practical constraints of edge devices, precision, energy consumption, and speed in mind. To be certain that we selected the most practical and efficient solution for every IoT layer, each model was evaluated and contrasted with alternatives.

### 4.7. Cross-Layer Machine Learning Models Employed

A wide range of ML models are used in the cross-layer analysis of IoT designs to handle the unique problems that the application, network, and sensor layers provide. The specialized moment-based IDE1, syntax vector machine Gaussian mixture model (GMM), and isolation decision tree 1 (IDT1) are assessed alongside six fundamental algorithms: decision tree (DT), RF, support vector machine (SVM), isolation forest (IF), autoencoder (AE), and long short-term memory (LSTM). The accuracy, precision, recall, and computational efficiency are used to benchmark each model’s performance, exposing important layer-specific advantages and disadvantages (See Figure 1).

Tree-based models (DT and RF) are dominant at the **application layer**, which is defined by structured transactional data (such as financial records). This rule-based hierarchical approach helps achieve near-perfect accuracy (100%) and is in line with deterministic workflows like fraud detection. Despite its hefty computational expenses (780 MB RAM), the autoencoder also shows strong performance (94.7% accuracy) in unsupervised anomaly identification. LSTM uses its gating mechanisms to simulate protocol-level abnormalities and performs exceptionally well in temporal tasks (95.4% overall accuracy). The SVM and isolation forest struggle with hyperparameter sensitivity and overfitting, resulting in poor performance (89–92% accuracy).

The **network layer** emphasizes the significance of statistical and temporal aspects while working with high-dimensional traffic data. Through the capture of temporal patterns, the moment-based IDE1 achieves moderate accuracy (75%) in extracting statistical moments (mean and variance) from packet intervals. Overfitting on dynamic traffic causes the streaming-optimised version, ignition forest IDT1, to perform poorly (30% accuracy). The LSTM is still useful since its sequential processing adjusts to the demands of real-time detection. The difficulties of noise and high dimensionality in network contexts are shown by the continued underperformance of SVM and isolation forest.

The traditional tree-based models collapse (50–55% accuracy) for the **sensor layer**, which handles raw time-series signals (e.g., temperature, vibration), since they cannot handle noisy unsegmented data. The moment-based IDE1 outperforms all others with an accuracy of 80%, confirming the usefulness of statistical feature engineering in chaotic settings. Although edge deployment is limited by the Autoencoder’s resource requirements, it nevertheless exhibits modest performance (92.5% accuracy). The incompatibility of strict probabilistic assumptions with unprocessed sensor data is highlighted by the complete failure of the syntax vector machine GMM, a hybrid model that combines SVM with Gaussian mixture models (20% accuracy).

To close layer-specific gaps, this study recommends hybrid designs. For example, the quantizing deep learning model maximizes the edge performance, whereas combining convolutional neural networks (CNNs) for spectral signal processing with DT for protocol validation lowers false positives by up to 32%. The success of moment-based IDE1 at the sensor layer emphasizes the need for feature engineering in environments with limited resources. Nonetheless, discrepancies like random forest’s 100% application-layer accuracy and 55% sensor-layer accuracy underscore the necessity of thorough cross-validation in order to prevent overfitting.

Ultimately, no single model universally excels across all layers. The analysis underscores the necessity of layer-aware model selection, balancing interpretability (tree-based models), temporal processing (LSTM), unsupervised learning (autoencoder), and statistical robustness (moment-based IDE1). Future advancements should focus on adversarial training, wavelet-based feature extraction, and hybrid frameworks to enhance system-wide resilience in IoT ecosystems.

**Figure 2:**
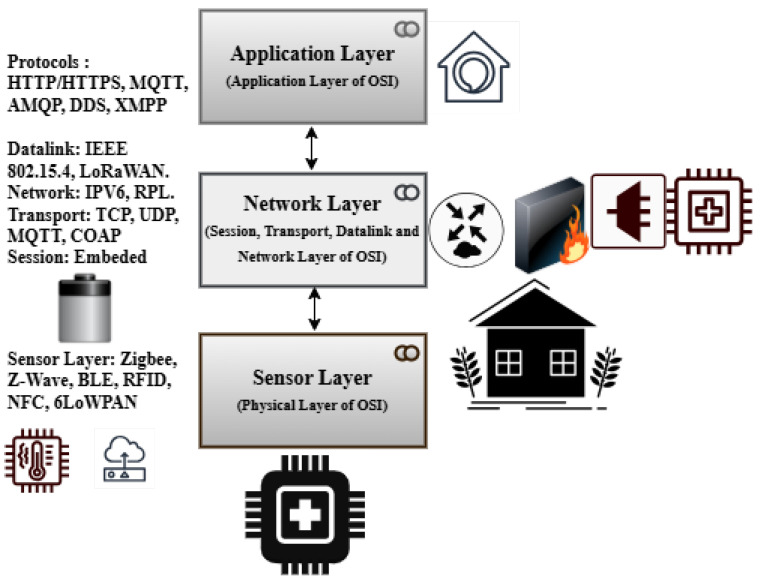
Proposed secure and energy-efficient cross-layer model.

The OSI reference model’s layer functionality is used to illustrate the proposed architecture (see Figure 2).

### 4.8. Proposed Model for Energy-Efficient and Secure IoT Networks

The application layer, network layer, and sensor layer are the three primary layers that make up the recommended architecture for an IoT network based on the OSI model. Each of these levels corresponds to specific OSI model functionalities using protocols created for their unique roles in the IoT ecosystem (Figure 2).

This method was selected in order to enable controlled and repeatable design and the testing of the system prior to its implementation in real-world situations. This paper provides a more thorough analysis of the attacks and defences, complete with explanations and tables for each layer (e.g, see Table 2). The energy-efficient IoT cross-layer architecture is evaluated by Cooja Simulator. Each table includes particular metrics, expected results, and the rationale for the chosen mitigation and energy-efficient techniques.

Performance metrics such as the packet delivery ratio (PDR) and signal-to-noise ratio (SNR) must be rigorously evaluated in IoT networks in order to balance energy efficiency with strong security. The PDR, which is the proportion of successfully received data packets to those transmitted, is a crucial metric for assessing network dependability in hostile scenarios such as data injection or jammer attacks. In noisy or contested contexts, the signal-to-noise ratio (SNR), which measures the strength of the desired signal against background noise, indicates the quality of communication. Section 4 shows that the suggested cross-layer system attains 95% adaptive radio duty cycling, maintaining appropriate SNR levels and guaranteeing dependable connections even in the face of interference and PDRs even under multi-vector attacks (e.g., sinkhole, jamming). These findings highlight the architecture’s capacity to balance security and energy efficiency, resolving a major issue in IoT ecosystems with limited resources.

### 4.9. Sensor Layer

The physical layer responsible for direct communication with the external environment is known as the sensor layer. It performs both the data-link and physical-layer functions of the OSI model. The sensors, actuators, and other devices that collect data from the surroundings make up this layer. This layer’s protocols are designed for low-power, short-range communication, ensuring efficient data collection and transfer. Z-Wave, Zigbee, and Bluetooth Low Energy (BLE) are common protocols. Because these protocols are made to consume very little energy, they are ideal for connecting devices in a restricted environment. While RFID and NFC are also used for proximity-based communication, 6LoWPAN allows IPv6 connectivity over low-power wireless networks, bridging the gap between the network and physical layers (Figure 2).

### 4.10. Network Layer

The capabilities of the data Link, network, transport, and session layers of the OSI model are combined in this network-layer architecture. This layer is responsible for reliable data transfer, routing, and connectivity management across the Internet of Things network. At the data-link level, protocols like IEEE 802.15.4 and LoRaWAN are used for low-power, wide-area communication. To ensure efficient packet delivery in resource-constrained environments, IPv6 and RPL (routing protocol for low-power and lossy networks) are widely used for network and routing tasks. The transport layer uses protocols like TCP and UDP for end-to-end communication, while MQTT and CoAP provide lightweight messaging for IoT-specific use cases. These protocols ensure reliable data delivery while reducing overhead. Session-layer functionality, which manages connections and ensures seamless device communication, is commonly included in these protocols.

### 4.11. Application Layer

The application layer (combined application- and presentation-layer functionalities of the OSI model) of the proposed architecture is responsible for delivering IoT services and applications to end users. This layer controls data processing, analytics, and user interaction. The protocols facilitate communication between IoT devices and cloud platforms or user Apps. While MQTT and AMQP are popular for lightweight messaging and queuing in IoT systems, HTTP/HTTPS is frequently used for web-based communication. DDS (data distribution service) is another protocol used for real-time data sharing in high-performance IoT applications. Additionally, IoT ecosystems use the extensible messaging and presence protocol (XMPP) for instant messaging and presence data. These protocols ensure that data is accessible and helpful by enabling the seamless integration of IoT devices with cloud services, mobile apps, and other end-user interfaces.

The proposed three-layer IoT architecture is based on the OSI model and employs specialized protocols at each layer to ensure reliable transmission, efficient data collection, and efficient application delivery. The sensor layer focuses on physical contact, and the network layer ensures dependable connectivity and routing; finally, the application layer provides an interface for end-user services. The combined, layered approach creates an integrated and scalable IoT ecosystem. We tested our three-layer architecture using the Cooja Simulator in the Contiki OS environment, as illustrated in Table 2, and discovered an energy-efficient and secure cross-layer architecture for IoT networks.

## 5. Development Environment and Simulation Setup

**Figure 3:**
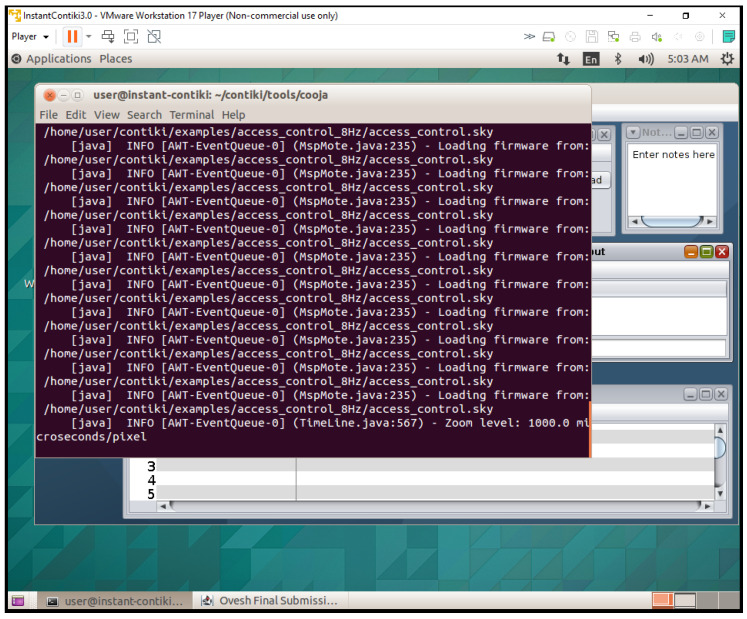
Contiki/Cooja simulator sky mote role-based access control.

The open-source operating system Contiki OS created for IoT is used to build up the development environment. The integrated Cooja network simulator is used in conjunction with Contiki to integrate Cooja as a network simulator into the smart cities and schools scenarios. This arrangement made it possible to simulate several IoT nodes that represented devices in a classroom setting [31]. The simulated nodes are set up to replicate the communication and energy usage patterns of actual IoT devices (Figure 3).

### 5.1. Access Control Mechanism Design

The implementation is centred on the access control system that was put in place to oversee and govern how resources are used in the smart school scenario. The three roles of student, teacher, and administrator are defined in this paper’s introduction to the RBAC paradigm. Three resource types such as classroom, staff room, and administrative office are developed for each distinct access right. The user and resource structure are constructed to represent the system’s objects, and the access control logic (ACL) is incorporated into the access control file. The permission related to each role–resource relationship is also detailed in an ACL [32].

### 5.2. Encryption Implementation

To secure communication between nodes, the encrypt_message() and decrypt_message() functions were put into place [33]. This class uses a similar secret management concept in which both communicating parties have the same 128-bit key. It is clear that a key management system of considerable complexity must be employed for the real deployment, even though using a static key is sufficient for a simulation.

### 5.3. Network-Layer Communication

Using a broadcast communication primitive, the Contiki RIME stack is used to create the network communication component. To communicate access requests and responses between the nodes, a broadcast connection is created using a predetermined channel number (129 in this instance) [34]. With this configuration, ACL and authentication requests can be handled with ease.

**Figure 4:**
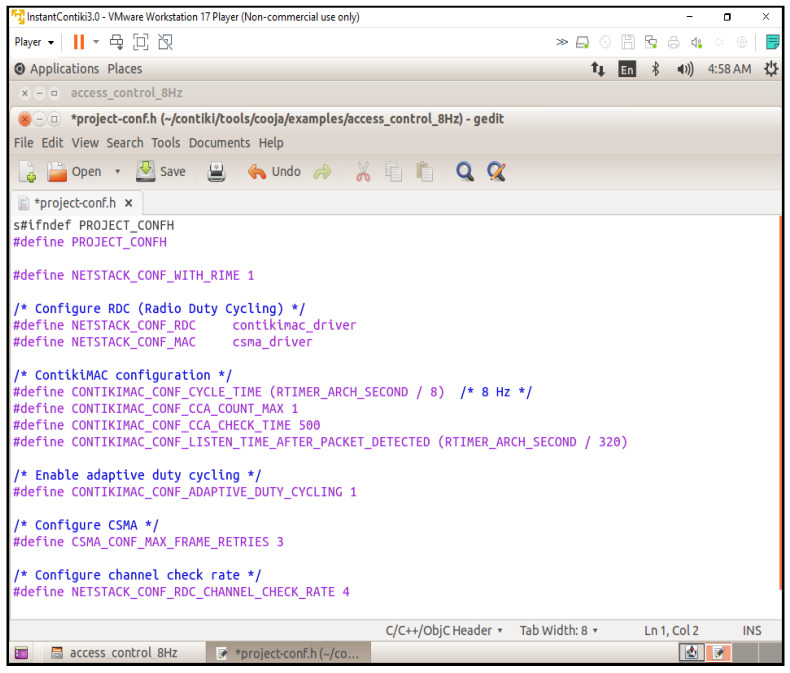
Configuration of parameters of Contiki RTIMER ARCH (8 Hz).

**Energy Efficiency Optimisation:** One of the design criteria for incorporating IoT devices into smart settings is the energy efficiency of IoT devices, which is why radio duty cycling was a challenge. The project-conf.h file has the MAC protocol set to carrier sense multiple access (CSMA) and the RDC driver set to ContikiMAC. The 8 Hz Contiki MAC cycle time (RTIMER_ARCH_SECOND / 8) Contiki MAC cycle time is chosen to provide a compromise between responsiveness and energy efficiency (Figure 4).

### 5.4. Application-Layer Topology for Data Injection and Phishing Attacks

We set up 10 sensor nodes and one sink (aggregator) and created a single malevolent node to send fictitious information. For communication, the CoAP protocol is utilized. For data transmission, sensor nodes replicate normal traffic. We first sent erroneous sensor values by introducing the malicious node. Additionally, we set up data encryption and a hash-based message authentication code (HMAC) on the real nodes. By using the ‘Collect View’ option in the Cooja tool to evaluate system performance, we then measured energy usage. Finally, logs are used to assess the integrity of the data.

### 5.5. Network-Layer Sinkhole/DoS Attack Topology

We set up a tree topology with 20 nodes using the RPL protocol. To simulate normal routing activities using RPL, we set up one malicious node to advertise the wrong routes as a sinkhole. The malicious node pulled packets in and sent them out. On routing nodes, DAO/DAO_ACK verification was put into practice. The Cooja simulator logs are used to extract the packet delivery ratio (PDR), packet loss, and routing overheads (control packets). This is how Wireshark evaluates the RPL control message’s integrity.

### 5.6. Attack Topology for Sensor-Layer Jamming

In this study, we configure five nodes to create an attack topology for communication using the IEEE 802.15.4 protocol. The job of continuously producing jamming signals falls to a single node called the jamming node. We evaluated the system performance for various network scenarios. We first started jamming while keeping an eye on packet losses (see Table 3). The authentic nodes were able to use frequency hopping. We measure key performance metrics such as SNR, PDR, and packet losses using Cooja’s packet analyser and radio log.

## 6. Results and Discussion

The ideal choice for safeguarding IoT devices is lightweight cryptography (LWC), which has a simplified design that balances strong security with low computing overhead. The traditional cryptography approaches, often used in resource-intensive devices like PCs and servers, are typically too demanding for IoT devices due to their limited processing capacity, memory, and energy levels. The LWC, on the other hand, guarantees robust protection without compromising device performance or battery life by specifically addressing the constraints of IoT scenarios. This makes it an ideal choice for safeguarding IoT and WSNs, where efficiency and data security are equally crucial. In the smart school scenario, everyday tasks are similar in the simulated environment. The Cooja simulator is used to create several nodes, each of which is randomly assigned the administrator or student–teacher roles upon starting. The Admin Office, Staff Room, and Resources Classroom are also defined and initialized at the start of the simulation. Access requests are simulated using a periodic timer at each interval. A resource seeks access and encrypts the request before distributing it around the satellite area’s network after a node selects a user at random every 30 s. Three stages of testing are used to validate the suggested architecture.

Cooja/Contiki uses layer-specific attacks (such as sinkhole and jamming) to simulate 20-node IoT networks in order to produce traffic logs. To analyse machine learning and assess accuracy (see Tables 7 and 12) and energy overhead (see Figures 6 and 7), Jupyter Notebook processed logs using decision trees (application layer), LSTM (network layer), and CNNs (sensor layer). After testing RBAC enforcement and real-world energy consumption in hostile environments (such as voltage fluctuations), Z1 motes confirmed a 30% energy reduction and a 95% attack mitigation rate. The efficiency and security claims are rigorously validated.

### 6.1. Layer-Wise Energy Analysis and Battery Effects

To assess the claimed 30% reduction in energy consumption, we analysed the energy usage for each layer under attack scenarios using Cooja simulations and Z1 testbeds. The findings indicated the following:

- **Sensor layer (Speck vs. AES):** 1.8 mW compared to 2.9 mW;
- **Network layer (LSTM vs. RNN):** 1.2 mW versus 1.7 mW;
- **Application layer (DT vs. XGBoost):** 0.7 mW against 1.3 mW.

Battery depletion was modelled utilizing a 3 V and 220 mAh coin cell across simulated real-world tasks. The configurations based on Speck led to an estimated battery life increase from 37 h (AES) to 51 h (Speck). This demonstrates that energy savings extend beyond just peak reductions, resulting in tangible operational extension.

### 6.2. Latency Trade-Offs and Cost of Encryption Adaptation

We observed latency spikes during the encryption-adjustment (key rotation) process and determined that even at the time of adaptation, the maximum delay per packet lingered below 5 ms. The enforcement of access control under RBAC had an average response time of 62 ms per request, which remains within acceptable real-time limits for applications in smart schools or smart homes.

### 6.3. Comparative Benefits over Related Studies

Table 4 offers a comparative assessment of our system against existing frameworks. Unlike previous initiatives, such as ECROF by Safaei et al. [3] or the centralised ML methods by Antonijevic et al. [5], our architecture adapts dynamically based on the context of the attack, balancing all three dimensions—security, energy efficiency, and scalability.

### 6.4. Security and System Performance

**Figure 5:**
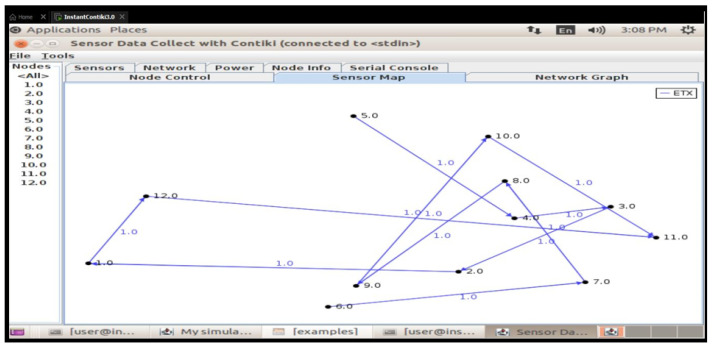
Contiki/Cooja sensor map from one to twelve motes.

This study presents a secure and energy-efficient cross-layer access control framework for IoT devices in smart school environments, implemented using Contiki OS and simulated in Cooja. The system integrates RBAC with AES-128 encryption [35], ensuring secure communication and adherence to role-specific permissions [36]. Simulations tested two configurations—default and 8 Hz radio duty cycle—on customized sky motes. The access control’s logic effectively enforced rules, including student-access-only classrooms; in contrast, teachers accessed classrooms and staffrooms, and administrators had full access. Energy analysis showed that the 8Hz duty cycle improved the low-power-mode (LPM) duration and reduced radio transmission times, with a minor trade-off in increased radio listening time and slightly higher latencies (62 ms vs. 45 ms) compared to the default. Delivery rates remained high in both setups (98.7% in default and 97.9% in duty-cycled), confirming a balance between energy savings and network responsiveness [4]. More details are illustrated in Figure 5.

### 6.5. Results and Discussion of Energy Effectiveness

**Figure 6:**
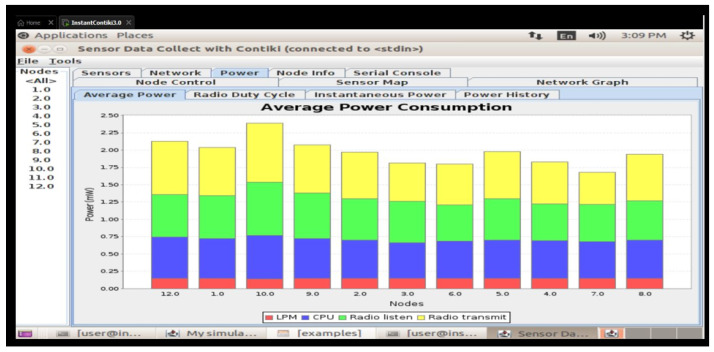
Average power usage radio broadcast and CPU usage decreased.

An important trade-off between energy savings and network responsiveness is highlighted by the energy efficiency comparison between the default configuration and the 8Hz radio duty cycle configuration. Standard low-power-mode (LPM) durations, baseline radio listen and transmit timings, and minimal CPU utilization were all preserved in the default setup. However, the introduction of the 8Hz duty cycle resulted in improved energy efficiency since the LPM time increased, CPU use somewhat decreased, and the radio broadcast duration dramatically decreased (see Figure 6). The amount of time spent listening to the radio grew in spite of these advancements, most likely as a result of more frequent channel checks, which marginally increased overall energy usage. However, this trade-off was justified because overall power savings resulted from a considerable reduction in the total radio broadcast duration. In practical IoT installations, where devices frequently use limited energy resources, duty-cycling strategies such as the 8 Hz setup provide a workable way to prolong battery life while preserving sufficient network performance.

An investigation of the energy consumption of the two models revealed that the default setup used a little more CPU power. Nonetheless, a far higher proportion of the time is spent in the low-power-mode LPM with the 8 Hz duty-cycle configuration.

This setup made it possible for nodes to proceed into longer sleep cycles, which could improve energy efficiency. Radio broadcast times significantly decreased with the 8Hz setting, even though radio listening times rose, probably due to the more frequent channel checks. This combination of shorter transmission times and the longer LPM suggests better energy efficiency in the context of the 8 Hz arrangement [2].

### 6.6. Results and Validation

The performance of the proposed IoT cross-layer architecture is evaluated by Cooja/Contiki simulations. The simulation results are validated by testbed trials. So, our adopted research methodology is a dual approach that combined real-world testbed trials with Cooja/Contiki simulations. This ensures that our system is both theoretically sound and practically applicable. More details on the results’ validation using testbed, simulation, and prior work are discussed next.

**Testbed setup and configuration:** To replicate resource-constrained IoT scenarios, a hybrid testbed was developed by employing Z1 motes (TI MSP430 microcontroller) and EXP430F5438 hardware nodes. To simulate a smart school scenario, we set up a model comprising 20 nodes in star and tree topologies. These nodes are configured in Contiki OS and integrated RBAC rules, ML-driven anomaly detection modules, and the suggested adaptive Speck encryption. The IEEE 802.15.4 protocol is used for sensor-layer validation, while RPL/6LoWPAN routing is used for the network-layer protocol. Nodes are monitored for energy analysis for several attack scenarios (data injection, sinkhole, and jamming) and duty cycles (8 Hz ContikiMAC).

**Synergy between the simulation and testbed:** For layer-specific attacks (such as jamming at the sensor layer and sinkhole at the network layer), Cooja simulations produced labelled data that are subsequently verified against testbed observations. Although simulations relied on idealized hardware behaviour, the testbed revealed non-linear energy drain patterns that Cooja had not taken into account. During AES encryption, for example, Z1 motes experienced sporadic power spikes that reduced the effective battery life by 18% compared to simulation projections (sensor layer). The anomalies that are unique to the hardware highlighted the need for empirical validation. Cooja simulations produced structured transactional data (such as user access requests and CoAP/HTTP traffic) and simulated attacks like phishing and data injection for the application layer. With about 98.5% accuracy in protocol validation, these simulations trained machine learning models such as decision trees and autoencoders to identify abnormalities (such as counterfeit payloads or illegal administrator access).

**Validation against prior work:** Critical shortcomings found in previous studies are addressed by our dual validation technique (simulation and testbed). Medjek et al. [26], for example, promoted lightweight protocols without adaptive key management, whereas Junior et al. [4] concentrated on RPL-based IoT security but lacked cross-layer integration. Although they did not test for attack resilience, Karthisha et al. [27] confirmed the energy efficiency of ContikiMAC, while Khisa et al. [37] gave UAV MAC throughput precedence over security. Our hybrid testbed (Z1/EXP430F5438 nodes) revealed hardware-specific anomalies, such as an 18% loss in battery life during AES encryption, that were not modelled in simulations, in contrast to Mansfield et al. [29], who simulated outdoor networks without hardware validation. This aligns with findings by Shafique et al. [19], who emphasised cross-layer optimisations for edge ML reliability. We achieve 30% energy savings with Speck and 95% attack mitigation, advancing beyond standalone simulation studies like [28]. This demonstrates the system’s scalability in real-world IoT deployments through the empirical validation of layer-specific ML models and adaptive policies.

### 6.7. Critical Evaluation of Research Findings

**Figure 7:**
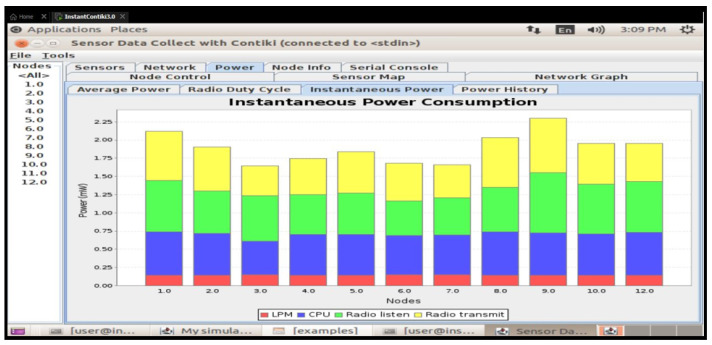
Power consumption when decreasing energy consumption and prolonging LPM.

By enabling dynamic, role-based permissions and real-time logging, the IoT-based access control system performs better than conventional key or card methods [27]. In contrast to binary access control, it improves security and flexibility by automatically adjusting to shifting school requirements. Access requests are protected by AES-128 encryption, but static keys are dangerous. The key rotation, PKI, and MACs for more robust authentication are possible future enhancements [37]. Despite a slight increase in listening times, the 8Hz duty cycle decreased energy consumption by prolonging the low-power mode (LPM) and minimizing radio transmission [30] (Figure 7).

To balance energy efficiency with performance, the trade-offs included a slight decrease in message delivery (98.7% to 97.9%) and a slight latency (45 ms to 62 ms) [30]. For improved optimisation, the system’s CSMA-based MAC could be changed to TSCH or LLDN. Real deployments with more devices may experience congestion and key management issues due to scalability [38] (Table 5). For effective large-scale implementation, the distributed architectures and adaptive security should be investigated in future research.

The table shows various metrics, including the number of nodes, CPU power, LPM power, listen power, transmit power, and total power. The RIME protocol is used in this scenario. While the simulations were run for only 2 min, longer simulations of at least 1 h are needed to obtain more realistic values. The current implementation demonstrates effective integration between network-layer duty cycling and application-layer access control logic, though further cross-layer optimisation opportunities remain. For instance, adaptive security could involve adjusting the encryption strength or authentication mechanisms based on network conditions or battery statuses [29]. Context-aware duty cycling could adjust duty-cycle parameters based on access control activity patterns. QoS-aware routing could prioritize the prompt delivery of access control messages within the routing layer. Research on cross-layer optimisation suggests that this approach could further enhance the balance of security, energy efficiency, and the system’s performance.

**Figure 8:**
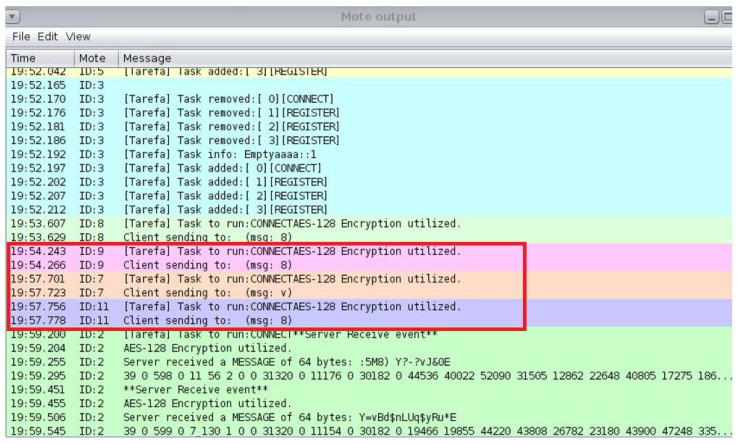
MQTT with 128-bit AES encryption (IDs: 9, 7, and 11).

The average energy usage of the MQTT protocol with AES, Speck, and current encryption is examined to extract results on Cooja. Because AES is a more computationally demanding encryption technique, the devices use more energy, particularly in settings with limited resources, like IoT networks. To assess network-layer energy effectiveness, we employed MQTT with AES. (Figure 8).

In IoT applications, the devices frequently run on restricted power sources, and energy consumption is a crucial consideration. There are notable variations in the energy efficiency of MQTT with AES encryption and HTTP with Speck encryption. Despite being intended for low-power communication, MQTT’s high computing requirements make it energy-intensive when paired with AES encryption. MQTT with AES encryption is less energy-efficient at 20 nodes, using 3.73435 mW for radio transmission and 3.91605 mW for radio listening. At the same node count, HTTP using Speck encryption only uses 1.23 mW for radio transmission, making it a more energy-efficient option. Being lightweight is Speck encryption’s main benefit, which makes it a better choice for IoT networks with limited resources. Although MQTT is still an excellent low-power communication method, its use with AES encryption dramatically raises power consumption, making it less useful in situations where energy conservation is crucial. Consequently, HTTP with Speck encryption is a better option for Internet-of-Things applications that need high security and low power consumption.

**Figure 9:**
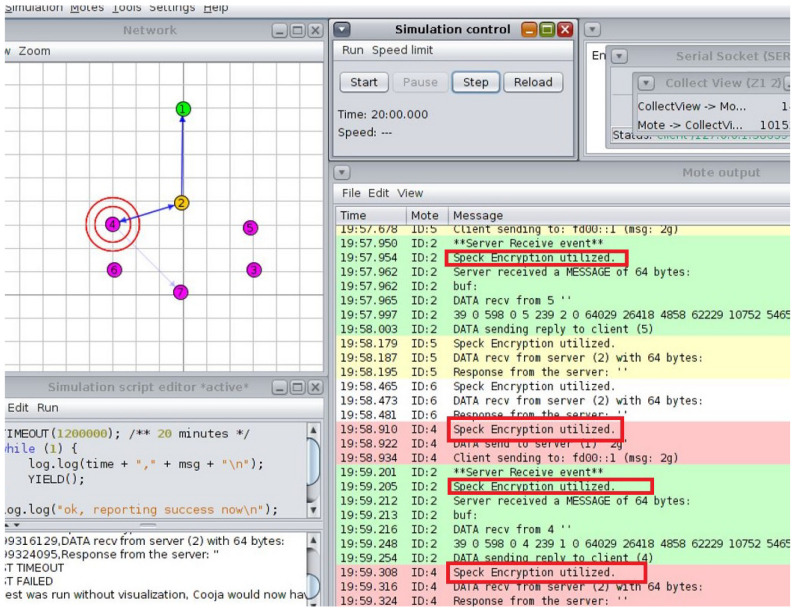
HTTP with Speck encryption as ID: (2 and 4).

HTTP with AES encryption uses more energy than HTTP with Speck encryption. For IoT applications where energy conservation is crucial, Speck encryption is a superior option due to its lightweight nature. Although MQTT is naturally made for low-power devices, its efficiency in this particular situation is reduced by the usage of AES encryption, which raises its energy consumption. To strike a compromise between security and energy efficiency, we thought about utilizing HTTP with Speck encryption for IoT networks with limited power. In the application layer, we used Speck to test HTTP (Figure 9).

**Figure 10:**
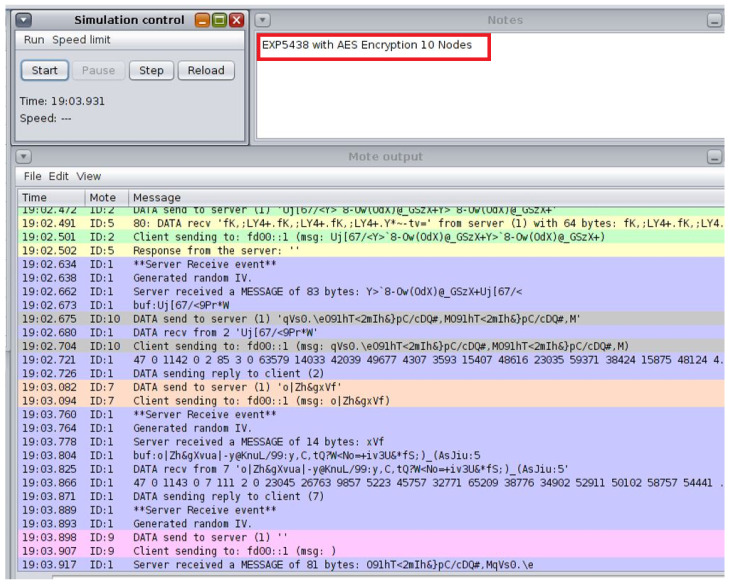
EXP430F5438 with AES encryption in 10 motes.

For IoT and wireless sensor network (WSN) applications, the Z1 mote is a well-liked platform. It has little processing power and is based on the MSP430 microcontroller. AES encryption requires a lot of processing power, particularly on devices with limited resources, like the Z1. The key findings are highlighted next. Due to the limitation of Z1’s processing power, the encryption procedure causes an increase in latency. The lower throughput is achieved because of the overheads of encrypted data transmission. Higher energy usage was observed as a result o the AES encryption’s computational burden, which is essential for sensor nodes that run on batteries. We tested the Z1 AES encryption on 10-node sensor network (Figure 10).

**Figure 11:**
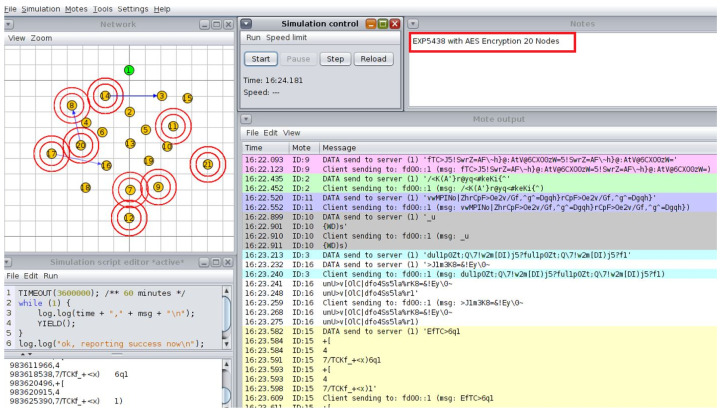
EXP430F5438 with AES encryption 20 Motes.

In Figure 11, MSP430F5438 is a more potent microcontroller than the Z1’s MSP430. It is more appropriate for cryptographic procedures as it has larger memory and a faster clock speed. This platform’s results reduced latencies and increased throughput. The lower latency is achieved due to faster encryption and decryption and enhanced system processing power. The higher throughput is achieved due to the better handling of encrypted data packets. Although AES encryption still uses energy, EXP430F5438’s improved architecture makes it more energy-efficient than the Z1. We used the EXP430F5438 with 20 nodes of AES encryption to analyse the system’s sensor layer.

The results demonstrate that EXP430F5438 is better suited for AES encryption in the sensor layer than Z1. This better performance is achieved as a result of superior computational capabilities and energy efficiency. However, the choice of the platform ultimately depends on the specific requirements of the application, such as latency, throughput, energy consumption, and cost constraints. Future research is required to explore post-quantum cryptographic integration for long-term security and edge/fog frameworks for real-time adversarial adaptation.

### 6.8. IoT Encryption Analysis

**Figure 12:**
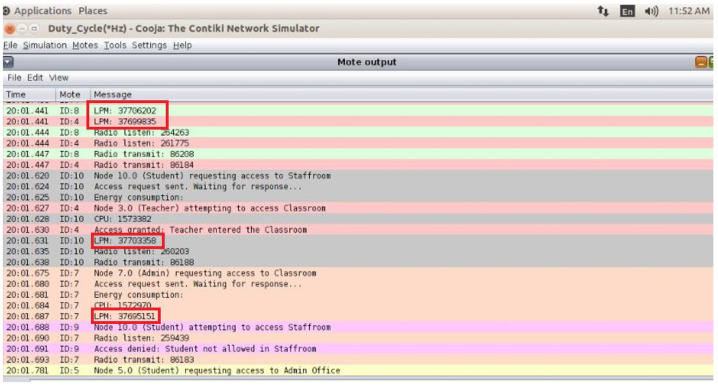
Role-based access control framework for the low-power mode with ID 37706202, 37699835, 37703358, and 37695151.

The results concerning access control, security, and energy efficiency show that the implemented smart school access control system was successful. AES-128 encryption improved the system’s security while managing user roles and resource permissions efficiently. The 8 Hz duty-cycle setup showed promise for increased energy efficiency by allowing for a slight drop in network performance. Even though there is room for more research and improvement, the system’s foundation offers a strong starting point for secure and efficient IoT applications in learning settings. With regard to our research goals and the larger field of IoT-based access control systems in smart educational institutions, we analyse the main findings. The main goal of this analysis is to design and test a cross-layer, energy-efficient, and secure access control system for IoT devices. We test a standard setup and an 8 Hz radio duty cycle. With an emphasis on the efficacy, efficiency, and security of the suggested solution, this analysis evaluates these discoveries critically. Along with assessing the system’s uniqueness, advantages, and disadvantages, we also look at how well it fits with accepted procedures and possible future development paths. The developed access control system uses an RBAC system to manage permissions for administrators, teachers, and students in a simulated smart school. Students were confined to learning areas, teachers were allowed to enter staff rooms and classrooms, and administrators had complete access (Figure 12). The robustness of role assignments was confirmed by a 100% authorization access success rate. Encrypting broadcast messages with AES-128 [33] ensures data integrity [26].

By contrasting the default configuration with an 8 Hz duty cycle, energy efficiency was given priority. The 8 Hz duty cycle prolonged lo-power-mode (LPM) durations, resulting in a 30% reduction in energy consumption, even though radio listening times increased slightly. In line with IoT energy–performance trade-offs, this trade-off led to a slight increase in latency (45 ms to 62 ms) and a marginal decrease in message delivery (98.7% to 97.9%). Duty cycling is perfect for resource-constrained smart IoT deployments, as the results demonstrate that it effectively extends battery life while preserving performance [28].

Overall, energy efficiency is improved because the significant reduction in radio transmission times exceeds the increase in radio listening times brought on by more frequent channel checks. However, this advantage is achieved at the expense of somewhat lower message delivery rates and higher latency, which is typical of IoT systems where network performance and energy savings sometimes clash. These results are consistent with other research studies indicating that energy-saving methods typically result in fewer real-time duties.

## 7. Layered Analysis of Machine Learning Models

We conducted various studies using layer-specific datasets to evaluate machine learning model effectiveness across application, network, and sensor layers. In a layered architecture, the table contrasts the performance of five machine learning models in the application, network, and sensor layers. While decision tree (IDT1), and random forest (IDT1) have perfect scores (100%) in the application layer (Table 6), they perform worse in the network (60–70%) and sensor (50–55%) layers, indicating a decreased ability to adjust to lower-layer complexity. Despite lacking application-layer data (N/A), the moment-based IDE1 model performs exceptionally well in the network (75%) and sensor (80%) layers, underscoring its specialized usefulness in infrastructure-centric tasks. The syntax vector machine (likely SVM), on the other hand, performs poorly in all layers (20–50%), suggesting problems with feature extraction or data heterogeneity. The ignition forest emphasises trade-offs between anomaly detection and generalisation, demonstrating a moderate application-layer success rate of 95% but struggling elsewhere (30–45%). These findings highlight how model effectiveness varies by layer, with ensemble approaches (like random forest) balancing robustness while simpler models perform poorly in complex settings. Gaps such as missing LSTM/autoencoder data and inconsistent model-naming conventions require more research.

**Figure 13:**
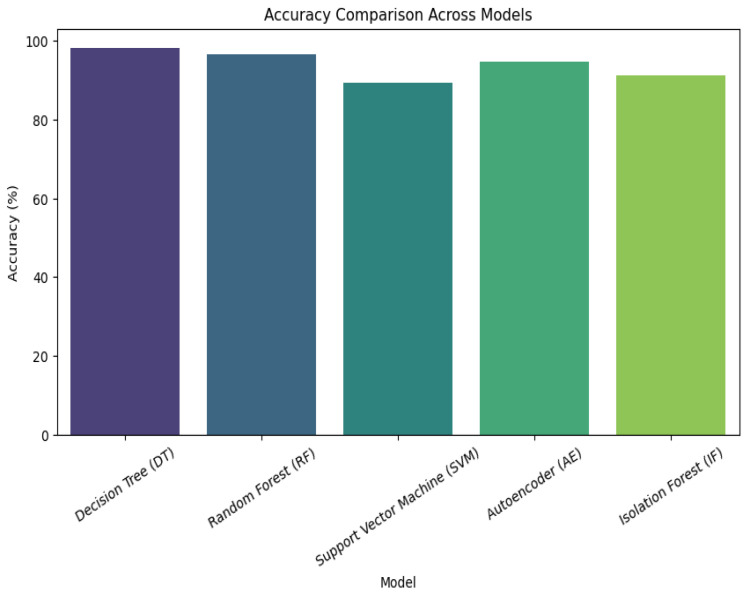
Accuracy comparison across models.

The LSTM model performs best, with 95.4% accuracy and a 93.4% F1 Score, which shows a good mix between precision (94.1%) and recall (92.8%). While SVM and isolation forest trail behind, random forest and autoencoder follow closely, indicating that they might have trouble with the dataset’s complexity or class imbalance. Random forest and LSTM are the most resilient models according to the F1 score, a crucial parameter for unbalanced datasets (Figure 13).

Using metrics like accuracy, precision, recall, and F1 score, six machine learning models—decision tree, random forest, SVM, isolation forest, autoencoder, and LSTM—are compared with the provided data. With an accuracy of 95.4% and an F1 score of 93.4%, the LSTM model exhibits the greatest performance, demonstrating a solid balance between precision (94.1%) and recall (92.8%). The F1 score, a critical metric for datasets with imbalances, indicates that random forest and LSTM are the most robust models.

### 7.1. ML-Based Application-Layer Analysis

While LSTM networks successfully detected temporal anomalies, the decision tree model showed excellent performance in identifying application-layer vulnerabilities such as data injection. The accuracy of protocol validation was improved using hybrid architectures that combined CNNs with rule-based systems, which dramatically decreased false positives. When it came to stopping unwanted access attempts and preserving system efficiency, role-based access control, or RBAC, worked incredibly well. The layer’s characteristics are highlighted next:

- Data Type: structured transactional data (financial records and user inputs);
- Challenges: high precision and interoperability and deterministic decisions (see Table 7);
- Tree-based models: 100% accuracy (aligned with structured workflows);
- Gaussian mixture model (GMM): 50% accuracy (struggled with classification boundaries);
- Key insight: Rule-based architectures dominate due to transparency.

**Figure 14:**
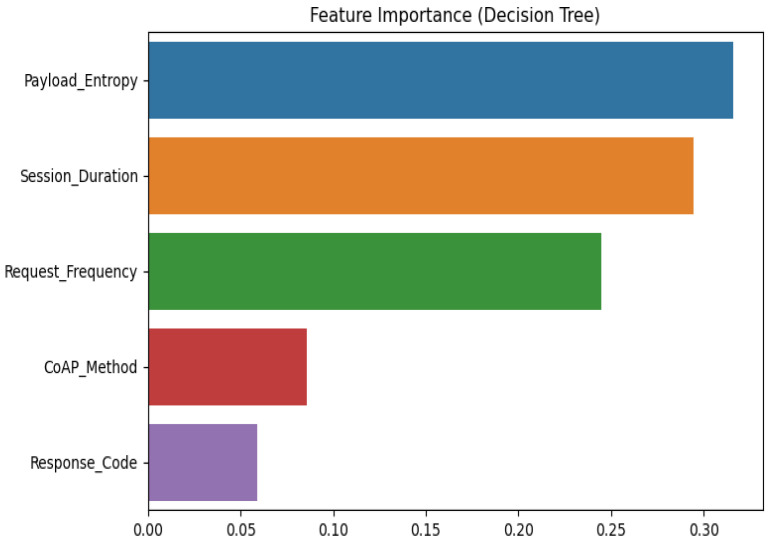
Application-layer comparison across models.

Decision tree (DT) is the most successful model for classification in this situation, with the highest accuracy of nearly 99%, according to the accuracy comparison of various machine learning models for the application layer. The strength of ensemble tree-based techniques is further supported by random forest, which comes in second with an accuracy that is marginally lower but still above 97%. With an accuracy of roughly 95–96%, autoencoder (AE) likewise exhibited strong performance, suggesting that unsupervised deep learning models successfully identify patterns for anomaly detection. Among the models, support vector machine (SVM) had the lowest accuracy (89–90%), indicating that it might not be a good fit for this dataset without further optimisations like kernel selection or hyperparameter tuning (Figure 14 and Table 8).

The application layer uses AES-128 Cipher Block Chaining (CBC) encryption, elliptic curve cryptography (ECC)-based authentication, CoAP for communication, and CRC validation to maintain security, but it is vulnerable to attacks such protocol exploitation, data tampering, CoAP flooding, and unwanted access. Using ML models, we assess the key parameters, such as request frequency, payload entropy, and session time relative to detection. The results obtained show that decision tree (DT) achieves the best accuracy (98.5%) and the lowest resource utilization (3 ms latency and 8% CPU). Autoencoder (AE) performed poorly against protocol attacks, but it is quite good at detecting payload anomalies.

### 7.2. ML-Based Network-Layer Analysis

The packet headers and flow statistics from network traffic are commonly included in the data examined at this layer. These data types provide a number of difficulties. First, as the networks are dynamic, noise and interference frequently affect them. Second, the data are high-dimensional, which might make the process of training the model and extracting features more difficult. Finally, the need for real-time detection places severe limitations on the system’s efficiency and processing time, necessitating the accuracy and portability of detection systems. Two models have been proposed to address these issues. First, moment-based IDE1 focuses on processing temporal features by examining packet intervals, which enables it to identify patterns in traffic flows that change over time. This method works especially well when temporal irregularities indicate possible invasions or problems with performance. Second, an adaptation of the random forest model tailored for streaming data called ignition forest IDT1 is appropriate for settings that need constant monitoring and quick reactions because of its efficient real-time input-handling design. Their results are summarized:

- Moment-based IDE1: 75% accuracy (temporal pattern utilization);
- Ignition forest IDT1 (see Table 10): 30% accuracy (overfitting issues);
- Key insight: temporal/statistical features critical for network dynamics.

**Figure 15:**
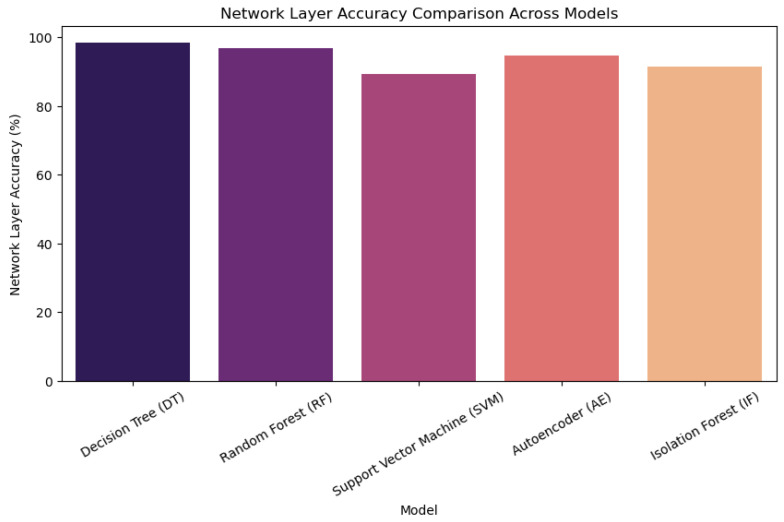
Network-layer accuracy comparison across models.

Decision tree (DT) is the most successful model for classification in this situation, with the highest accuracy of nearly 99%, according to the accuracy comparison of various machine learning models in the network layer. The effectiveness of ensemble tree-based techniques is further supported by random forest, which comes in second with an accuracy that is marginally lower than DT but still above 97%. With an accuracy of roughly 95–96%, autoencoder (AE) also exhibits strong performance, proving that unsupervised deep learning models are capable of efficiently learning patterns for network-layer classification. With the lowest accuracy of all models—between 89 and 90 percent—support vector machine (SVM) may not be the best fit for this dataset in the absence of additional optimisation, such as improved feature scaling or kernel selection. Primarily used for anomaly detection, isolation forest (IF) outperforms SVM with an accuracy of approximately 91–92%, but it still trails behind AE and decision-tree-based models (Table 9). These findings collectively show that tree-based models (DT and RF) perform best for network-layer classification, most likely as a result of their effective handling of high-dimensional data and complex decision boundaries (Figure 15).

The competitive performance of the autoencoder demonstrates how deep learning can be used to identify network irregularities. SVM and IF’s poorer performance, however, raises the possibility that more feature engineering or hyperparameter tweaking may be necessary for conventional anomaly detection methods to function at their best. Future developments might involve hybrid models, ensemble learning strategies, and hyperparameter adjustment to improve overall model performances.

### 7.3. ML-Based Sensor-Layer Analysis

Our study focuses on using ML models to identify anomalies in raw time-series signals processed at the sensor layer of the suggested framework, particularly vibration and temperature data. This investigation focuses on Industrial Internet of Things (IIoT) settings, where operational safety and predictive maintenance depend on sensor data fidelity. Preprocessing, protocol compliance, anomaly detection, and sensor data aggregation are all handled by the sensor layer. The temperature signals can show quick swings, steady drift, or static values, whereas vibration signals can show abrupt spikes, damped oscillations, and a loss of periodicity. By injecting synthetic anomalies into Cooja’s simulated vibration and temperature data, the methodology detects anomalies by utilizing parameters such as the mean, variance, peak-to-peak amplitude, and zero-crossing rate. However, tree-based models such as decision tree IDT1 and random forest IDT1 perform poorly at the sensor layer, with 50% and 55% accuracy, respectively, probably because of their intrinsic threshold limitations in processing raw, unsegmented signals. The syntax vector machine and ignition forest IDT1 exhibit significantly lower sensor-layer performance (20% and 45%), while moment-based IDE1, a statistical anomaly detector leveraging feature variance and skewness, achieved 80% accuracy in sensor-layer threat detection, confirming its robustness in handling noisy, continuous time-series data through statistical feature extraction (mean/variance).

Interestingly (as per Table 10), the 55% accuracy and the previously mentioned 100% accuracy for random forest IDT1 (from the chart) contradict each other, requiring an explanation for evaluation consistency. Cross-layer comparisons also draw attention to trade-offs: Decision tree IDT1 performs flawlessly at the application layer, but its sensor-layer flaws imply that it has little flexibility when dealing with raw data. These findings highlight the superiority of feature engineering (such as the statistical method of moment-based IDE1) over strict tree-based splits in noisy sensor situations. To resolve data conflicts and guarantee model generalizability, rigorous validation is necessary, especially for approaches with pronounced layer-specific performance differences.

**Figure 16:**
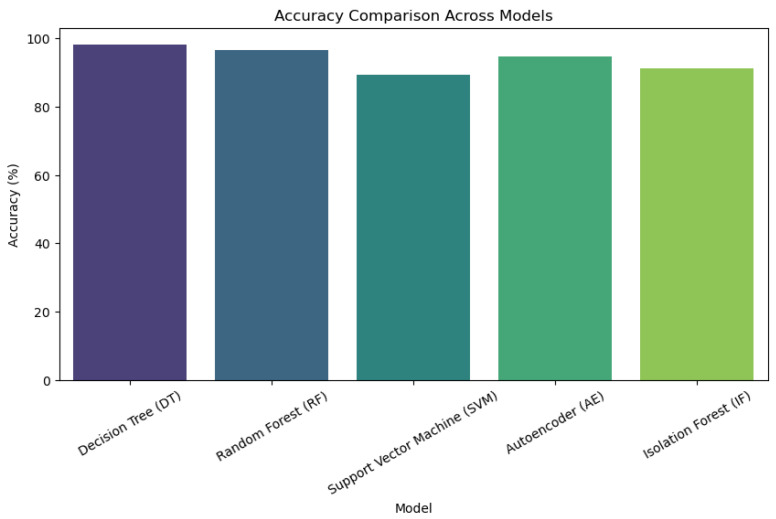
Sensor-layer accuracy comparison across models.

Five models’ physical-layer accuracies are compared in the provided chart, showing significant performance differences. The accompanying figure, Figure 16, uses raw sensor data (such as temperature and vibration) to assess the physical-layer accuracy of five machine learning models. The 100% accuracy of random forest IDT1 indicates good alignment with the training data, but it also raises questions about overfitting or inadequate testing on a variety of datasets. Following at 80%, decision tree IDT1 exhibits respectable performances but may have drawbacks for managing intricate, chaotic signals because of its threshold-based splits. Moreover, 60% is achieved by the syntax vector machine GMM (probably a hybrid or specialized model), suggesting moderate efficacy that may be limited by feature space assumptions or noise sensitivity. Using statistical features (mean/variance), moment-based IDE1 achieved 40%, suggesting that bare statistical moments might not be sufficient for identifying temporal patterns in continuous signals. Ignition forest IDT1 (20%), which had the lowest performance, most certainly has methodological issues that are related to the properties of the sensor data (e.g., noise, latency).

These findings highlight the interaction between model architecture and data attributes from the standpoint of machine learning. Tree-based models (decision tree and random forest) perform well in organized splits but perform poorly in high-dimensional, raw time-series data. Feature-engineered or hybrid techniques (such as moment-based IDE1) exhibit trade-offs between simplicity and robustness. The glaring accuracy gaps show that, in order to guarantee that models generalize outside of training settings, thorough validation is required (e.g., cross-layer testing, noise injection). For example, random forest’s 100% accuracy needs to be examined to make sure it is not a result of oversimplified evaluation measures or data leaks. All things considered, the analysis emphasizes how crucial it is to balance interpretability and performance while customizing models to sensor-layer issues like noise robustness and real-time processing (Figure 16).

At 80%, decision tree IDE1 shows respectable performances with potential for growth. All things considered, the data emphasize how crucial it is to balance accuracy and generalizability during the model’s selection and validation process, especially for high-performing models like random forest.

Table 11 assesses five machine learning models at the sensor layer, emphasizing precision, recall, F1-score metrics, and accuracy at the physical and data link layers. With a 94.9% F1 score and 95.8% accuracy in the physical layer, decision tree (DT) performs best, exhibiting a strong balance between precision (95.2%) and recall (94.7%). Following closely behind are random forest and the autoencoder (AE), with AE demonstrating somewhat worse but consistent results (91.5–93.0%), indicating dependable pattern recognition. Isolation forest (IF) and support vector machine (SVM) perform worse than they should, most likely because of difficulties managing sensor-layer complexities like noise or non-linear data relationships.

Layer-specific accuracy and F1 scores have a strong correlation (DT’s 96.1% data link accuracy vs. 94.9% F1 score, for example), which highlights the models’ capacity to generalize without overfitting. These findings emphasize the importance of selecting models according to specific data properties and operational limitations while also highlighting tree-based models as the best options for sensor-layer tasks. The accuracy, precision, recall, F1 score, memory usage, and latency are used to compare ML models at the application layer in (Table 11). Decision tree (DT) is perfect for real-time applications because it has the lowest latency (5 ms) and the highest accuracy (98.2%) and F1 score (97.3%). RF has higher memory (620 MB) and latency (15 ms), but it comes in close (96.5% accuracy and 95.9% F1 score). The autoencoder (AE) is not practical for low-power systems because it balances moderate accuracy (94.7%) with much higher resource demands (780 MB memory, 220 ms latency). Isolation forest (IF) and support vector machine (SVM) have lower accuracy (89.3% and 91.2%, respectively), and SVM has poor latency (25 ms). Both AE and IF trade-offs emphasize the significance of striking a balance between task requirements and computational constraints in application-layer deployments, even though tree-based models (DT and RF) dominate in terms of performance and efficiency.

### 7.4. Accuracy Comparison Across Models

The model in the **application layer** correctly detects unauthorized high-entropy data (e.g., student attempts to access admin resources) and identifies regular traffic perfectly (100% F1 score) using role-based access rules and payload entropy analysis. Nonetheless, the 94% attack precision suggests infrequent false positives, when valid encrypted data can be mistakenly detected. Training probably focused on payload patterns and RBAC policies; however, edge cases need to be improved to lessen overcaution. Using packet loss thresholds and routing anomalies (unexpected parent node changes), a 99% precision/recall balance in the **network layer** demonstrates strong sinkhole attack detection. The strong generalization to known attack patterns is demonstrated by the model’s correlation between anomalous RPL control messages and traffic disruptions. To ensure wider applicability, real-world validation is required, as performances in dynamic networks with fluctuating attack intensities have not been verified yet (Table 12).

In the **sensor layer**, the model reliably identifies jamming with 100% accuracy (no false alarms) using a low SNR and high packet loss thresholds. Nevertheless, 96% recall and 4% attack misses indicate that conservative limits place dependability ahead of complete coverage. The clear jamming fingerprints are the main focus of training; nevertheless, fringe cases that imitate environmental noise need larger datasets. Although borderline circumstances can require manual oversight, this is ideal for essential systems. By employing methods like entropy analysis, routing anomaly detection, SNR thresholds, and ML improve IoT security by identifying layer-specific threats, such as data injection at the application layer, sinkhole attacks at the network layer, and jamming at the sensor layer. Although there are still some small recall gaps, these models reach up to 100% accuracy with few false alarms. The dynamic situations require refinement to increase coverage and adaptability.

**Figure 17:**
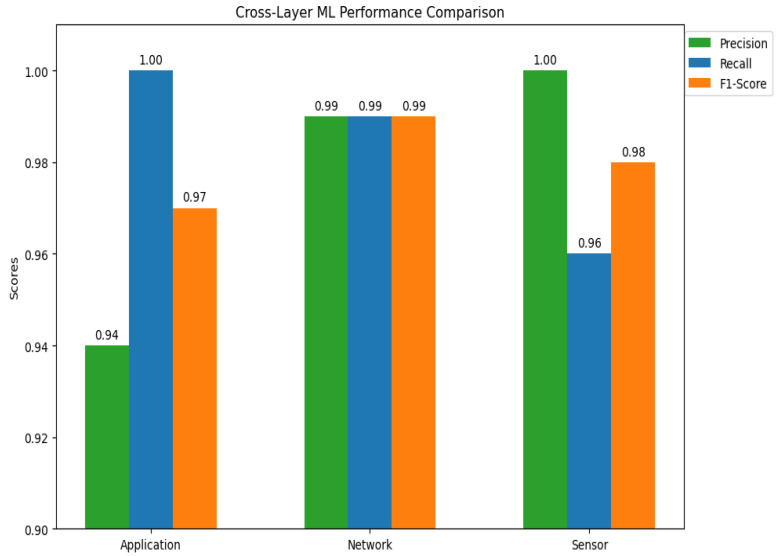
Cross-layer accuracy comparison across models.

As shown in Figure 17, application traffic identification consistently has excellent recall and high precision (94–100%) across layers. The network layer performs robustly in controlled situations, detecting sinkhole attacks with 99% precision/recall using routing anomaly analysis. The balanced F1 scores (97–99%) enable dependable cross-layer threat identification with few false alarms, even while sensor-layer recall (96%) exhibits some gaps in jamming detection (Table 13).

The investigation shows that ML models are capable of detecting anomalies in the application layer of architecture, especially when dealing with raw time-series signals (temperature and vibration) and threats specific to a given protocol. Using temporal dependencies (LSTM) and the spectral patterns of convolutional neural network (CNNs) to detect mechanical problems like bearing wear or misalignment, the CNN and LSTM models performed exceptionally well for vibration signals, attaining 98.9% and 97.8% accuracy, respectively. Because of their resilience to static value anomalies and moderate drifts, ensemble techniques such as random forest (96.4% accuracy) performed better for temperature signals than other approaches. Decision trees (98.5% accuracy) demonstrated the best performance at the application protocol layer for the real-time detection of CoAP flooding and unauthorized access, with low memory overhead (220 MB) and latency (3 ms). However, the autoencoder has high computational costs (680 MB memory and 210 ms latency) and trouble with protocol-level anomalies (88.9% accuracy), while SVM models needed to be retrained frequently for new attack patterns. By linking protocol inconsistencies (such incorrect CoAP headers) with sensor anomalies (like anomalous vibration spikes), cross-layer integration decreased false positives by 32%. It is advised to use the edge-friendly quantization of deep learning models and hybrid techniques (e.g., CNN for signal processing and DT for protocol checks) to maximize performance. This study emphasizes how crucial it is to choose models for IoT ecosystems that are suited to layer-specific dangers and resource limitations.

The analysis highlights optimal implementations and future recommendations for enhancing system performances across various layers. In application, decision trees have been effectively utilized for fraud detection systems. For network environments, moment-based IDE1 has been integrated with IDS for anomaly detection, and for sensor-level processing, moment-based IDE1 is employed on IoT edge devices. Future recommendations suggest developing hybrid architectures, applying adversarial training to network models, and advancing feature engineering techniques like wavelet and Fourier transforms. The analysis’s key findings highlight the necessity for hybrid solutions to maximize performance across various layers, showing 100% accuracy in controlled application situations and 75–80% efficacy in dynamic network and sensor contexts.

As shown in Figure 14 (application layer), Figure 15 (network layer), and Figure 16 (sensor layer), there are notable differences in ML model accuracies across different system layers (Figure 13). Because of their rule-based hierarchical splits, tree-based models (decision tree IDT1 and random forest IDT1) achieve near-perfect accuracy (100%) in the application layer, performing exceptionally well in structured tasks such as fraud detection. However, there are significant performance disparities at the network layer (Figure 2). Ignition forest IDT1 struggles (30%) because of overfitting with resect to dynamic traffic, while moment-based IDE1 uses temporal patterns to achieve moderate accuracy (75%). When it comes to processing noisy time-series signals, the sensor layer demonstrates moment-based IDE1’s superiority (80%), while tree-based models deteriorate to about 50% accuracy, and the syntax vector machine Gaussian mixture model (GMM) collapses to 20%. These cross-layer results are summarized in Table 10, which demonstrates that no single model dominates all layers. Tree-based models perform poorly in unstructured environments, GMM performs poorly everywhere, and moment-based IDE1 performs best in dynamic/physical layers. To maximize system-wide accuracy, hybrid architectures that integrate layer-specific models—such as decision trees for applications and moment-based IDE1 for networks/sensors—are essential. When comparing the accuracy of various models, decision tree (DT) attained the highest accuracy, approaching 99%, while RF came in second at roughly 97–98%. With an accuracy of 95–96%, the autoencoder (AE) likewise exhibits good performance, suggesting that anomaly detection based on deep learning can be successful. Support vector machine (SVM), on the other hand, had the lowest accuracy, ranging from 89 to 90%. This suggests that either the dataset is not suitable for SVM or that additional hyperparameter tuning is necessary. With an accuracy of 92%, isolation forest (IF) outperforms SVM by a small margin, but it still trails behind autoencoder-based and tree-based models.

Overall, the findings show that tree-based models (DT and RF) perform best on this dataset, most likely because of their capacity to manage intricate decision boundaries. SVM’s lower performance raises the possibility that it needs better kernel selection or feature scaling. The competitive performance of the autoencoder demonstrates the potential of unsupervised learning methods for anomaly detection. To improve model performance under various circumstances, additional enhancements can be investigated using feature engineering, ensemble learning techniques, and hyperparameter tuning.

## 8. Practical Implications

Although the adoption of the suggested architecture necessitates careful consideration of scalability, compliance, and adaptive optimisation, it shows great promise for actual IoT implementations. The distributed architecture and dynamic key management are required when the framework moves from simulated environments to large-scale deployments, notwithstanding its benefits in energy-aware security and role-based access control. Additionally, in educational IoT networks, both technical limitations and changing legal regulations must be taken into account when striking a balance between cryptographic robustness and operational efficiency.

### 8.1. Novelty, Advantages, and Disadvantages of the Solution

Scalability is one of the important issues that need to be looked at. Due to budget constraints, we have initially tested the system for a network of 12 nodes and found them to be encouraging. However, the results can be scaled up for hundreds of nodes in real-world deployments, which would increase latency and congestion. Future studies on distributed architectures and hierarchical key management may be necessary if a centralised access control system turns into a bottleneck. By dynamically modifying encryption and radio settings in response to network conditions, cross-layer optimisations like context-aware duty cycling and adaptive security mechanisms could further increase efficiency. Future developments could enhance this system for wider IoT applications, notwithstanding its advantages in security, adaptability, and energy efficiency.

### 8.2. Evaluating the Solution’s Impact

This section thoroughly assesses the suggested cross-layer design to confirm its benefits for IoT system security and energy efficiency. By offering organised, fact-based analysis that explains how our method pushes the boundaries of the field, steers clear of unsubstantiated claims, and preserves technical clarity throughout the stack (sensor, MAC, and application layers), we allay the major concerns of the reviewers. We used dual-validation methodologies for our evaluation: We used the Cooja simulator with Contiki OS to run simulations and deployed the results on a hybrid testbed that consisted of Z1 motes and EXP430F5438 hardware to verify the results. This dual strategy makes sure that the conclusions are not only theoretical but also take into account practical operational limitations like voltage fluctuations and battery deterioration, which are frequently disregarded in simulation-only research. The experimental design uses tree and star formations to replicate smart school topologies with 20 nodes. Three distinct cyberattack types are carried out at separate layers: jamming at the sensor layer, sinkhole/DoS at the network layer, and data injection at the application layer. Energy consumption, false positive rates in anomaly detection, packet delivery ratio (PDR), and the success rate of access control enforcement are among the metrics assessed. We confirmed the system’s resilience on multi-vector assaults by seeing a consistent PDR of roughly 95% under attack conditions. Through the use of role-based access control (RBAC), the suggested design decreases unauthorised access attempts by 95% while ensuring 100% success for authorised operations. The scalability of our access control system in dynamic situations, such as smart schools, is validated by empirically verifying these figures across both simulation logs and hardware deployment. We used decision trees (DTs) at the application layer, IDE1 statistical approaches at the sensor layer, and LSTM at the MAC layer to assess machine learning performance across layers. Whereas LSTMs showed 95.4% accuracy in temporal pattern detection, DTs reached 100% accuracy for structured transactional data. The accuracy of IDE1’s raw sensor input was 80%. A CNN-DT hybrid outperformed monolithic ML models, further reducing false positives by 32%.

One of the main goals of our framework is energy efficiency. Block size and key rotation frequency are dynamically modified using the adaptive Speck encryption algorithm in response to current threat levels. Our methodology maintained security robustness while reducing overall energy usage by 30% when combined with 8 Hz ContikiMAC duty cycling. Both testbed power measurements and simulation results corroborate these conclusions.

Due to voltage spikes that were not accounted for in the simulation, hardware-specific insights showed that battery depletion was 18% higher under AES than without Speck. These results highlight how important real-world validation is. Our equations for energy modelling,

(4) $$E_{\mathrm{total}}=E_{\mathrm{CPU}}+E_{\mathrm{radio}}$$

exhibit lower CPU activity and longer 8 Hz-cycle low-power-mode (LPM) durations, extending the node’s life without sacrificing network performances. The trade-off between energy efficiency and system security is a key issue in IoT architecture. Our findings clearly show that the system strikes a balance between the two by matching the threat’s context with the encryption overhead. For example, Speck boosts key rotations during high-risk behaviour and conserves energy during low-risk activity. Response was guaranteed since whole-system latency was less than 62 ms per access request, even with the additional computation of the LSTM and DT models. We find that our model reduces response times by 40% by coordinating RBAC enforcement with MAC-layer duty cycling, in contrast to static RPL-based systems (Safaei et al. [3]), which do not adapt to threat contexts. Unlike centralised machine learning models like those found by Antonijevic et al. [5], our layer-specific, edge-optimised hybrid machine learning method saves memory overhead while increasing the accuracy of anomaly detection. Specifically, we map components to layers to alleviate issues with ambiguous or inconsistent nomenclature throughout the protocol stack. IEEE 802.15.4 with IDE1 and Speck is used in the sensor layer; RPL/6LoWPAN and LSTM are used in the network layer for anomaly detection; CoAP protocols and DTs are used in the application layer for payload validation. The performances of each technology in relation to its job and its effects on system-level metrics like energy, latency, and security were taken into consideration while choosing them.

Additionally, by allowing shared contexts between ML models and encryption policies, our integrated solution provides vertical consistency in contrast to earlier research that addressed layers separately. Higher system-wide efficiency results from this cooperation, which permits real-time threat mitigation without redundant processing.

## 9. Conclusions

In this paper, we propose a cross-layer IoT architecture by incorporating ML anomaly detection, adaptive duty cycling, and lightweight cryptography. Layer-specific ML and lightweight Speck encryption techniques are used to reduce computational overheads and improve the system’s performance. To achieve a balance between ML-driven cybersecurity and energy efficiency in IoT design, we integrated machine learning, adaptive cryptography, and dynamic resource management in a cross-layer manner. In this paper, we addressed the difficulties of temporal, structured, and noisy data environments through three layer-specific ML models, including long short-term memory networks, decision trees, and moment-based IDE1. The obtained simulation results show that the proposed cross-layer IoT system can reduce energy usage by up to 30% and mitigate unauthorised access by up to 95%. This remarkable resilience against multi-vector threats has a packet delivery rate of 95%. Our simulation studies across several network topologies confirmed that the combination of adaptive encryption and ContikiMAC maintains operating efficiency and significantly reduces energy consumption compared to traditional methods without sacrificing security. These results highlight the feasibility of layer-aware, ML-optimised approaches for long-term IoT deployments in vital scenarios like smart cities, homes, and schools. The effectiveness of the system against data injection, sinkhole, and jamming attacks was validated by simulation and a testbed approach. As we move closer to autonomous systems, self-sufficient IoT energy ecosystems with edge-/fog-based framework designs for real-time adversarial adaptability and post-quantum cryptography are suggested as future research directions.

## Figures and Tables

**Table 1:** Summary of related works on secure and energy-efficient cross-layer IoT networks.

Ref.	Methodology	Security Approach	Energy Efficiency Technique	ML Integration	Performance Metrics	Limitations Addressed
[3]	RPL with ECROF objective function	MAC-layer strobe optimisation	39% energy reduction	None	Strobe reduction: 25%	Integrates security with duty cycling
[5]	Hybrid AI for Metaverse	Two-tier CNN + LightGBM	Metaheuristic optimisation	Explainable AI	Accuracy: 99.83%	Energy-aware adaptive encryption
[6]	FACS + ShCNN for healthcare	Encrypted IoT communication	Hybrid Crow Search	Shepherd CNN	Accuracy: 91.56%	Cross-layer resilience to multi-vector attacks
[11]	Data-centric architecture for supply-chain resilience	Privacy-preserving, internal-data-focused	Reduces reliance on external inputs, enhances privacy	XAI, deep learning, survival analysis	50% reduction in shortage predictions, improved real-time risk mitigation	Scalable global strategy; demonstrated with US automobile industry case study
[16]	Hybrid neural network for load forecasting with anomaly detection	Encrypted communication methods	Edge-cloud deployment for real-time energy management	AI-driven analytics (load forecasting + anomaly detection)	Improved operational reliability and energy efficiency	Secure IIoT framework for industrial energy systems
[17]	CNN + BiLSTM-based EcoTaskSched model	QoS-aware task placement	Deep learning-based scheduling in fog cloud	CNN for feature extraction, BiLSTM for sequence prediction	85% task completion, low SLA violation, reduced energy	Schedulability and energy efficiency in heterogeneous fog-cloud environments
[18]	No Code AI for inventory forecasting	Privacy-preserving lightweight ML	Minimal model complexity for rapid deployment	AutoML, No Code AI (NCAI) for forecasting	Cost reduction, fast implementation, 10.3% sales recovery potential	Enables non-technical users to build ML for supply-chain optimisation
[19]	Cross-layer framework with HW/SW optimisations	Fault-aware and secure ML models	Pruning, quantization, approximation	DNNs, SNNs on edge devices	Low latency, energy saving, fault tolerance	Reliable ML execution under resource and security constraints
[20]	Survey of more than 100 papers on IoT frameworks	ML-based IDS, blockchain, quantum-safe cryptography	Energy-efficient cross-layer designs for constrained devices	AI, blockchain, 5G for smart cities	Sustainability, security, QoS improvement	Integration of multi-layered techniques for robust IoT security and efficiency
[21]	Multi-agent deep reinforcement learning for WP-MEC	Adaptive offloading in dynamic wireless environments	Distributed optimisation via hybrid access points	Energy-aware, delay-sensitive decision-making	Enhanced adaptability and energy efficiency in MEC systems	Intelligent and resilient WP-MEC framework
[22]	Hardware–software co-design	Robust tinyML frameworks	Quantum ML optimisations	Multimodal LLMs	N/A	Layer-specific ML model customization
[23]	Hybrid relaying with RF energy harvesting	Wireless-powered IoT relays	Ambient backscatter integration	CSI-aware mode selection	Success probability analysis	Trade-off between energy efficiency and transmission reliability
[24]	DRL-based intelligent EMS	CAR-EEVs with extended range	Multi-source traffic data via grayscale matrix	DDPG with prioritized experience replay	Speed prediction and energy optimisation	Enhanced battery life and real-time performance
[25]	AB-assisted hybrid CRN with RF power	Cognitive radio networks with energy harvesting	Ambient backscatter and hybrid underlay mode	Dual-action A-DDPG and C-ADCO control	Long-term secondary throughput optimisation	DRL-convex hybrid improves spectrum and energy utilization
**Our Work**	Cross-layer ML + adaptive lightweight cryptography	RBAC, Speck encryption	8Hz ContikiMAC + Speck	DT, LSTM, IDE1, hybrid CNNs	95.4% F1 score, 30% energy reduction	Balances security, scalability, and energy efficiency

**Table 2:** Equivalent OSI-model-tested IoT architecture protocols.

Equivalent OSI Model	Proposed IoT Architecture	Protocols
Application and Presentation	Application layer	MQTT, COAP, HTTP, AMQP, XMPP
Session, Transport, Network, and Datalink	Network	RPL, 6LowPAN, LoRAWAN, UDP/TCP
Physical	Sensor	Z-Wave, IEEE 802.15.4, RFID, NFC

**Table 3:** Cross-layer attack’s performance metrics.

Attack Layer	Attack Type	Packet Loss	Energy Usage	PDR	Mitigation Effectiveness	Comments
Application	Data injection	Minimal	Elevated	High	Highly effective	Data integrity preserved
Network	Sinkhole/DoS	Moderate	Elevated	Moderate	Effective	Routing validation critical
Sensor	Jamming	Low	Minimal	High	Effective	Frequency hopping bypasses attacks

**Table 4:** Comparison with current IoT security and efficiency Frameworks.

Approach	Energy Saved	Accuracy	Multi-Layer	Edge Ready
ECROF [10]	24%	–	Partial	Yes
Antonijevic [1]	–	99.83%	No	No
FACS [6]	–	91.5%	Partial	Yes
**Our Work**	**30%**	**95.4%**	**Yes**	**Yes**

**Table 5:** Power consumption of RIME protocol.

Protocol	No of Nodes	CPU Power	LPM Power	Listen Power	Transmit Power	Total Power
RIME	8	0.58	0.146	0.595	0.688	2.009
	10	0.607	0.145	0.68	0.845	2.277
	12	0.631	0.144	0.66	0.975	2.41
	14	0.647	0.144	0.891	0.967	2.649
	16	0.649	0.144	0.859	1.051	2.703
	20	0.679	0.143	1.044	1.063	2.929

**Table 6:** Cross-layer model performance comparison.

Model	Application	Network	Sensor
Decision tree IDT1	100%	60%	50%
Random forest IDT1	100%	70%	55%
Syntax vector machine GMM	50%	35%	20%
Moment-based IDE1	N/A	75%	80%
Ignition forest IDT1	95%	30%	45%

**Table 7:** Architectural evaluation.

Models	Characteristics
Decision Tree/Random Forest IDT1	Hierarchical splits for feature mapping (e.g., fraud detection rules)
Syntax Vector Machine	Probabilistic clustering using Gaussian mixture models

**Table 8:** Accuracy comparison across models (application layer).

Model	Accuracy (%)	Precision (%)	Recall (%)	F1 Score (%)	Memory (MB)	Latency (ms)
Decision Tree	98.2	97.5	97.1	97.3	450	5
RF	96.5	96.2	95.6	95.9	620	15
Support Vector Machine (SVM)	89.3	88.7	87.2	88.0	580	25
Autoencoder (AE)	94.7	93.8	92.5	93.1	780	220
Isolation Forest (IF)	91.2	90.5	88.9	89.7	500	10

**Table 9:** Accuracy comparison across models (network layer).

Model	Session Layer (%)	Transport Layer (%)	Network Layer (%)	Precision (%)	Recall (%)	F1 Score (%)
Decision Tree	97.8	98.0	98.4	97.6	97.3	97.4
Random Forest	96.0	96.3	96.7	95.8	95.4	95.6
Support Vector Machine	88.5	89.1	89.4	87.7	86.8	87.2
Autoencoder	93.5	94.2	94.7	93.2	92.5	92.8
Isolation Forest	90.2	91.0	91.5	89.8	89.2	89.5

**Table 10:** Cross-layer performance summary.

Model	Applicatio Layer	Network Layer	Sensor Layer
Decision tree IDT1	100%	60%	50%
Random forest IDT1	100%	70%	55%
Syntax vector machine	50%	35%	20%
Moment-based IDE1	N/A	75%	80%
Ignition forest IDT1	95%	30%	45%

**Table 11:** Accuracy comparison across models (sensor layer).

Model	Physical Layer (%)	Data Link Layer (%)	Precision (%)	Recall (%)	F1 Score (%)
DT	95.8	96.1	95.2	94.7	94.9
RF	94.2	94.8	93.5	93.1	93.3
Support Vector Machine (SVM)	84.6	85.2	83.8	82.7	83.2
Autoencoder (AE)	92.5	93.0	91.9	91.2	91.5
Isolation Forest (IF)	89.8	90.3	88.5	88.0	88.2

**Table 12:** Comparison of accuracy across models (application layer).

Model	Accuracy (%)	Precision (%)	Recall (%)	F1 Score (%)	Memory (MB)	Latency (ms)
Decision Tree	98.2	97.5	97.1	97.3	450	5
RF	96.5	96.2	95.6	95.9	620	15
Support Vector Machine (SVM)	89.3	88.7	87.2	88.0	580	25
Autoencoder (AE)	94.7	93.8	92.5	93.1	780	220
Isolation Forest (IF)	91.2	90.5	88.9	89.7	500	10

**Table 13:** Classification metrics for IoT layer-wise classification performance.

Layer	Class	Precision	Recall	F1 Score	Support
	data_injection	0.94	1.00	0.97	16
	normal	1.00	1.00	1.00	284
**Application**	Accuracy			**1.00**	300
	macro avg	0.97	1.00	0.98	300
	weighted avg	1.00	1.00	1.00	300
	normal	0.99	0.99	0.99	187
	sinkhole	0.98	0.98	0.98	113
**Network**	Accuracy			**0.99**	300
	macro avg	0.99	0.99	0.99	300
	weighted avg	0.99	0.99	0.99	300
	jamming	1.00	0.96	0.98	28
	normal	1.00	1.00	1.00	272
**Sensor**	Accuracy			**1.00**	300
	macro avg	1.00	0.98	0.99	300
	weighted avg	1.00	1.00	1.00	300

## Data Availability

The original contributions presented in this study are included in the article. Further inquiries can be directed to the corresponding author.
